# *Thalassiosira pseudonana* growth phase determines gene expression and algicidal behavior of a new *Alteromonas macleodii* strain

**DOI:** 10.1128/mbio.00275-26

**Published:** 2026-03-23

**Authors:** David Wiener, Zinka Bartolek, Riley Dunklin, E. Virginia Armbrust

**Affiliations:** 1 School of Oceanography, University of Washington 7284, Seattle, Washington, USA; 2 Department of Science, Engineering and Math, Cypress College 17049, Cypress, California, USA; Oregon State University, Corvallis, Oregon, USA

**Keywords:** marine bacteria, algicidal bacteria, algal-bacterial interactions, diatoms, growth phase, phytoplankton

## Abstract

Phytoplankton-bacterium interactions are pivotal in marine ecosystems, influencing primary production and biogeochemical cycles. Diatoms, in particular, engage in diverse relationships with bacteria, ranging from mutualism to pathogenicity. However, the mechanisms governing the shift between these interactions and how they are shaped by host physiology and environmental context remain unclear. To address this, we investigated how the growth phase of the model diatom *Thalassiosira pseudonana* influences the behavior of an *Alteromonas macleodii* strain isolated from the Equatorial Pacific. We find that *A. macleodii* EP displays a differential algicidal impact on *T. pseudonana*, depending on the growth phase of the diatom, with the greatest effect occurring during the stationary phase. The algicidal effect can be amplified to impact all growth phases by the addition of an external organic carbon source to the co-cultures. Transcriptomic analysis revealed that *A. macleodii* EP shifts from motility-associated to growth-associated gene expression patterns in response to the diatom’s growth phase and co-culture duration. Bacterial cell-free exudate growth assays and fluorescence microscopy of co-cultures show a two-stage interaction model: initial bacterial swimming and exudate secretion induce diatom death, followed by bacterial aggregation around diatom debris. Comparative transcriptomics of *A. macleodii* with other algal hosts highlights host-specific bacterial responses, underscoring the context-dependent nature of these interactions. Together, these findings reveal how bacterial behavior and gene expression are modulated by host state and environmental cues, providing a molecular basis for the dynamic roles of diatom-bacterium interactions in shaping microbial community structure.

**IMPORTANCE:**

Diatoms are responsible for almost a quarter of global primary production, and their ecological roles are shaped by interactions with heterotrophic bacteria. These relationships can range from mutualistic to algicidal, with consequences for nutrient cycling and carbon export. Here, we show that the growth phase of the model diatom *Thalassiosira pseudonana* shapes its interaction with a newly isolated open-ocean strain of *Alteromonas macleodii*. Using transcriptomics and co-culture experiments, we demonstrate that bacterial gene expression dynamically shifts with host physiology, transitioning from motility and algicidal activity to aggregation and nutrient acquisition. Our findings reveal a two-stage interaction dynamic and highlight diatom growth phase as a critical, yet often overlooked, factor in determining the outcome of bacteria–algae interactions. By linking host physiology to bacterial behavioral shifts, this study provides new insights into how microscale dynamics can scale up to influence ocean productivity and biogeochemical cycling.

## INTRODUCTION

Phytoplankton exist in complex communities, sharing their microenvironment with diverse microbes, including heterotrophic bacteria (1). Phytoplankton-bacterium interactions can influence algal physiology as well as large-scale processes such as carbon export and nutrient cycling (2). Diatoms generate about 20% of global net primary production (3), and their ecological impact is shaped by interactions with marine bacteria that influence their growth, survival, and community composition (4). Diatom-bacterium interactions range from pathogenic to mutualistic (4–6). For example, certain bacteria provide diatoms with essential vitamins and growth-promoting compounds, while others release algicidal molecules that inhibit diatom growth or cause cell lysis. Transitions from mutualism to algicidal behavior within a given bacteria-algae pair have been demonstrated, driven by factors such as nutrient availability (7), co-culture duration (8–10), and community composition (11). In turn, diatoms can promote beneficial bacteria while suppressing opportunists through the secretion of compounds such as rosmarinic and azelaic acids (12, 13). Understanding the molecular mechanisms governing these interactions is crucial for elucidating the ecological role of diatoms and the potential impacts of small-scale interactions on larger biogeochemical processes.

*Alteromonas macleodii* is a marine heterotrophic bacterium known for its versatility in forming both beneficial and detrimental interactions, highlighting its dynamic role in marine ecosystems (7, 14–17). Notably, for a successful algicidal effect, some *A. macleodii* strains require physical attachment (7), while others rely solely on secreted compounds (15), demonstrating variable algicidal mechanisms. A recent study found that the *A. macleodii* behavior in co-culture with the model diatom *T. pseudonana* fluctuated from mutualistic to pathogenic with the addition of organic matter, which triggered bacterial movement toward the diatom and induced a protease-mediated algicidal effect (7). Yet, the molecular basis for the observed bacterial behavioral changes and how they adapt to exposure to diatoms in different physiological states remains hidden. Additionally, the *A. macleodii* strains tested in co-culture with diatoms were obtained from coastal waters, so it remains unknown whether open-ocean strains exhibit similar interaction dynamics (7, 14, 16).

The growth phase shapes the physiological status of unicellular organisms and is often broadly divided into two main stages: the exponential phase, characterized by active growth, and the stationary phase, in which cell division and death are balanced (18–20). Diatoms show growth phase-specific transcriptomic (21), proteomic (22), and metabolomic (23) profiles. As diatom cells progress through their growth phases, they secrete organic matter, which serves as a substrate for bacteria (20). These physiological changes of the diatom influence diatom-associated bacterial composition in laboratory settings and natural communities (23, 24). Most co-culture experiments begin with diatoms in their exponential growth phase, leaving it unclear how encountering diatoms in other growth phases influences bacterial molecular responses.

Here, we characterize the interaction between a new *A. macleodii* strain, recently isolated from the equatorial Pacific Ocean, and the model *T. pseudonana*. We found that this *A. macleodii* strain exhibits an algicidal effect that depends on the diatom’s growth phase and organic matter availability, and that the algicidal effect can be suppressed by a putative diatom defensive response. The new strain dynamically adjusted its transcriptional program over time in response to diatoms at different growth phases. Specifically, the *A. macleodii* strain downregulated genes related to motility (such as those involved in chemotaxis and flagellar assembly) and upregulated genes involved in cellular growth (including components of the translation machinery and ribosomal genes), depending on the diatom’s growth phase and the duration of the co-culture. A combination of fluorescence microscopy of co-cultures and bacteria-free filtrate treatments suggests a two-stage interaction model. In the first stage, bacterial cells swim and secrete exudates that induce diatom death, an effect that can be triggered either by the presence of diatoms or by bacterial growth on an alternative organic matter source. This is followed by a second stage, where bacterial cells aggregate around diatom debris. Our study shows that the host’s growth phase influences bacterial-algal interactions, with bacterial gene expression modulation shaping the interaction dynamics.

## RESULTS

### Characterization of the interaction between *A. macleodii* EP and *T. pseudonana* across different growth phases

**Fig 1:**
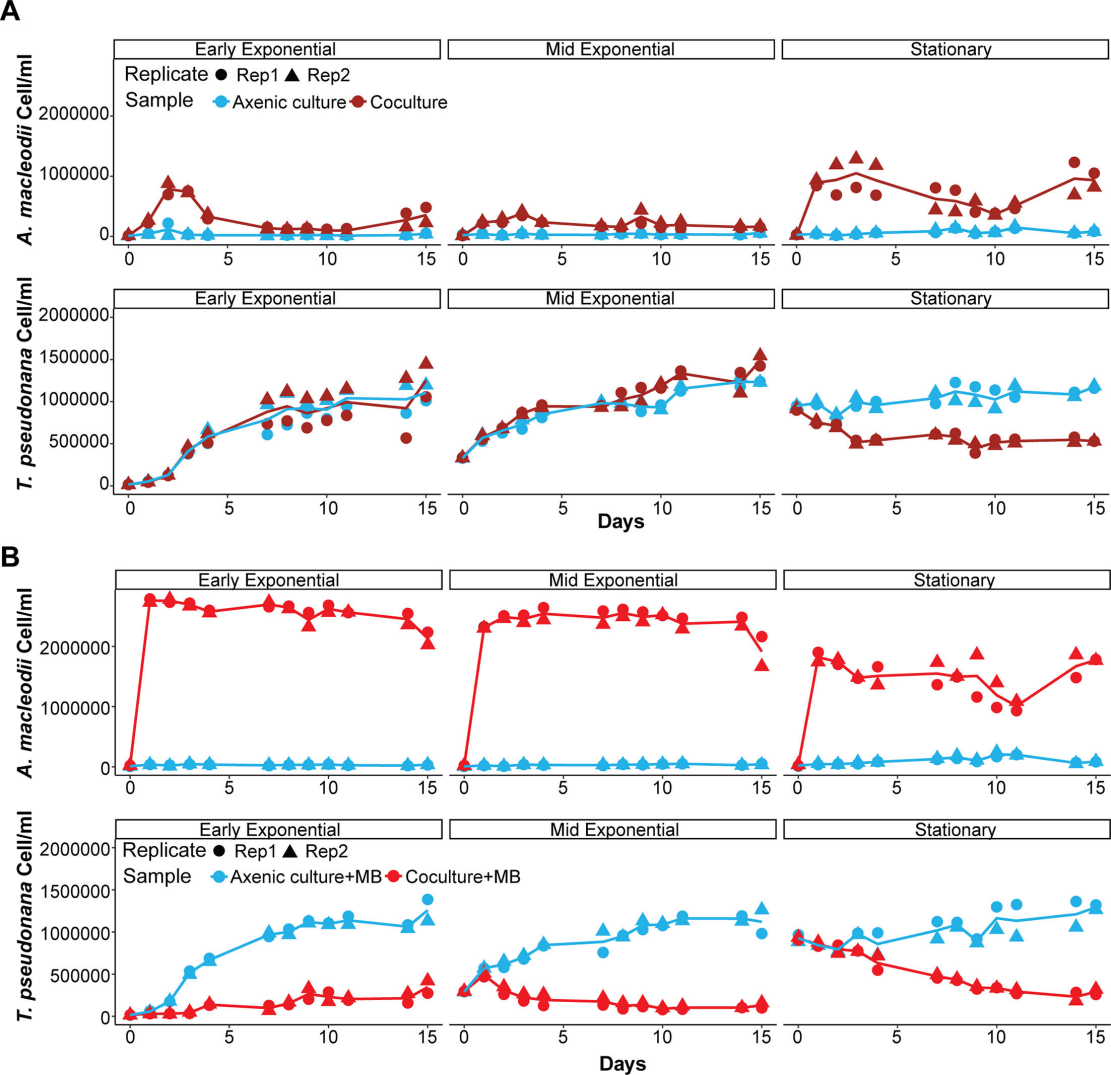
Initial culture conditions for *T. pseudonana* play a key role in determining interaction dynamics with *A. macleodii* EP. (**A**) Growth curves of bacterial cells (top) and diatom cells (bottom) when grown in co-culture (magenta symbols) or as mono-cultures (blue symbols); replicates are indicated with different symbol types (circles, triangles), with the line indicating the average of two biological replicates. Each facet corresponds to a different diatom growth phase at the start (*t* = 0 days) of the experiment (early exponential, mid-exponential, or stationary phase). (**B**) As in panel **A**, but with the addition of 2% (vol/vol) marine broth at the start of each experiment (red symbols).

We cultured and evaluated the physiological characteristics of a new *A. macleodii* isolate from seawater collected on a research cruise (Gradients 4) in the equatorial Pacific (0.5° N, 139.73° W, 15 m depth) on 5 December 2021. The new isolate was most closely related to two publicly available *A. macleodii* strains based on 16S ribosomal DNA sequence identity (98.8% to NR_037127 and 98.1% to NR_114053) (Fig. S1; Table S1). We designate this new isolate as *A. macleodii* EP. Although *A. macleodii* EP and *T. pseudonana* are not known to co-occur naturally, given the variety of interactions reported between *A. macleodii* and unicellular algae, we initiated co-cultures by adding the new *A. macleodii* isolate to *T. pseudonana* at early exponential, mid-exponential, or stationary phase without the addition of an external organic carbon source. We defined the *T. pseudonana* stationary phase as 7 days after the last cell doubling, when cell concentration reached ~800,000 cells/mL. The two exponential growth phases were defined relative to stationary phase: early exponential-phase cultures were at ~25,000 cells/mL, approximately five doubling events before reaching stationary-phase cell concentrations; mid-exponential/exponential-phase cultures were at ~200,000 cells/mL, two doubling events before reaching the stationary-phase cell concentration (Fig. 1A and B, lower panels). When the co-cultures were initiated with diatoms in early or mid-exponential phase, *A. macleodii* EP abundance increased over the first few days and then decreased in abundance, likely due to depletion of available organic carbon (Fig. 1A upper panel). In contrast, when *A. macleodii* EP was co-cultured with a stationary-phase *T. pseudonana* culture, *A. macleodii* EP reached significantly higher cell concentrations (ANOVA followed by *post hoc* pairwise comparisons with Bonferroni-adjusted *P*-values: early exponential–mid exponential = 1, early exponential–stationary = 0.0001, mid exponential–stationary < 0.0001, Table S5), presumably due to increased availability of organic carbon released by *T. pseudonana* over the growth cycle (Fig. 1A, upper panel). During the stationary phase co-culture, *A. macleodii* displayed an algicidal effect on *T. pseudonana,* resulting in a 0.3-fold decrease in final diatom abundance (Fig. 1A, lower panel). These results suggested that either stationary-phase *T. pseudonana* cells were particularly vulnerable to *A. macleodii* EP or that a sustained increase in *A. macleodii* EP abundance was required for the algicidal impact.

To evaluate whether stationary-phase *T. pseudonana* cells displayed enhanced sensitivity to *A. macleodii* EP, we controlled bacterial abundance in each set of co-cultures by adding 2% (vol/vol) marine broth (MB) as an external organic nutrient source. The addition of organic matter had no impact on the growth of *T. pseudonana* in axenic mono-cultures (Fig. 1B). With the addition of organic matter, *A. macleodii* EP reached high cell numbers in each co-culture and either prevented an increase in *T. pseudonana* abundance (early exponential) or resulted in a decrease in *T. pseudonana* abundance (mid-exponential and stationary phase) over time (Fig. 1B). A similar algicidal effect was observed if the organic carbon was added 4 days after the initiation of the co-culture (Fig. S2A). Two other bacteria*—Ruegaria pomeroyii* and *Sulfitobacter* sp.—did not display an algicidal impact under the same co-culture conditions, confirming that the observed *A. macleodii* EP algicidal effect was not an artifact of our culture conditions (Fig. S2B). Together, these results indicated that *T. pseudonana* was susceptible to the algicidal impact of *A. macleodii* EP regardless of the diatom growth phase. Additionally, the final concentration of *A. macleodii* EP in the supplemented co-cultures varied depending on the initial growth phase of *T. pseudonana*. When co-cultured with stationary-phase *T. pseudonana*, *A. macleodii* EP cell numbers were reduced by 0.66-fold by the 24-hour time point (Fig. S2C) compared to those co-cultured with exponential-phase *T. pseudonana*. Similarly, a 0.79-fold reduction was observed when MB was added 4 days after the initiation of late-exponential-phase *T. pseudonana* co-cultures (Fig. S2D). This latter result suggested that the diatom somehow suppressed the growth of *A. macleodii* EP, perhaps due to either a constitutive release of putative *A. macleodii* EP growth modulating compounds throughout the diatom growth cycle—the effect tied to diatom cell numbers—or to a differential release of these compounds during the diatom stationary phase.

### *T. pseudonana* limits *A. macleodii* EP growth

**Fig 2:**
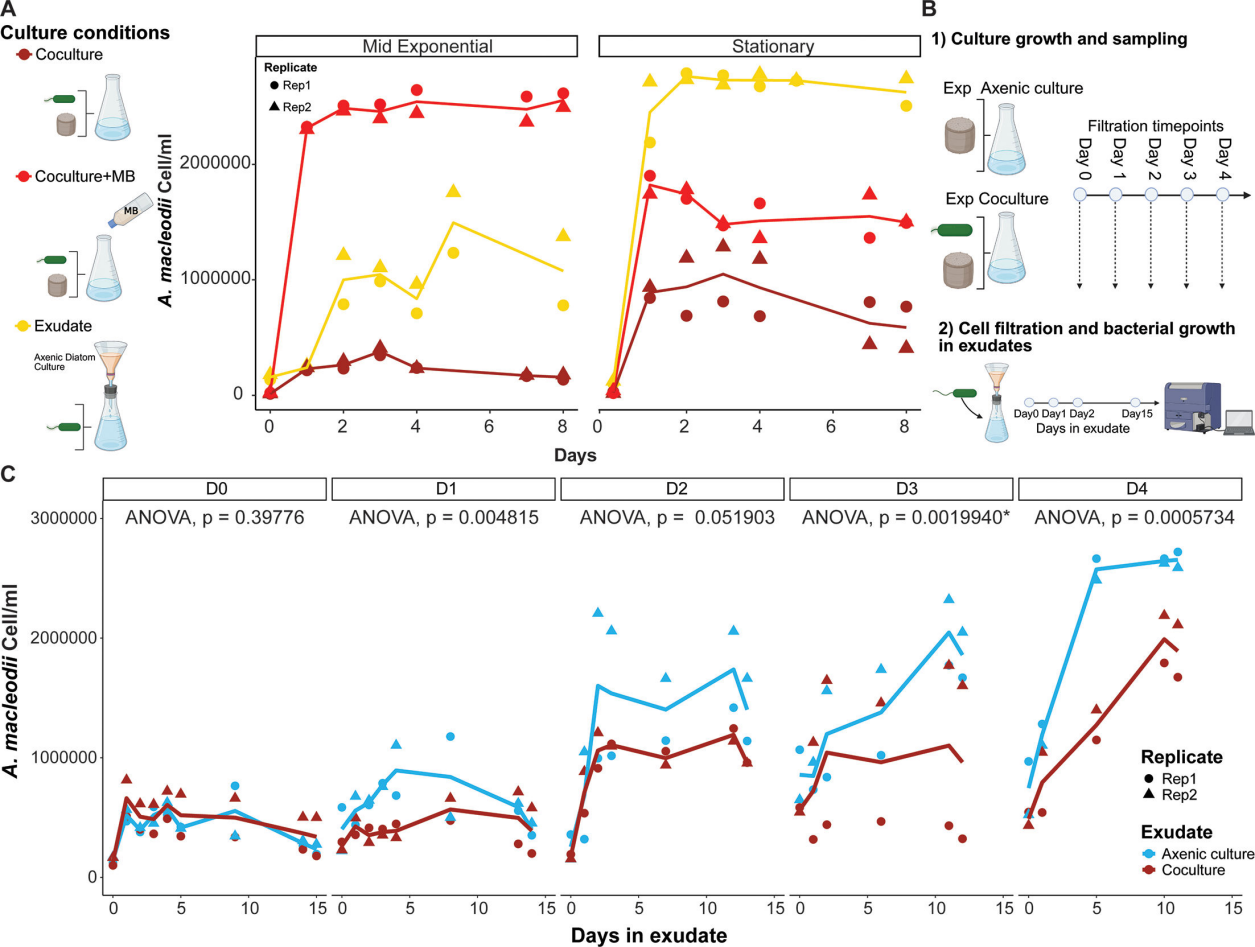
*T. pseudonana* imposes constraints on bacterial growth beyond nutrient limitation. (**A**) Scatter plot of bacterial cell counts measured by flow cytometry, with lines indicating the mean of two replicates. Cartoons represent the culture conditions in which bacterial growth was tested. Colors distinguish bacteria grown in co-culture (magenta), in co-culture plus marine broth (co-culture + MB; red), and with exudates from axenic *T. pseudonana* cultures (yellow). Each condition was tested with both exponential and stationary *T. pseudonana* cultures, and results are shown in separate facets. (**B**) Schematic representation of axenic culture and co-culture filtrate experiments. Cell-free exudates were collected every 24 h from both axenic and co-culture conditions. Identical amounts of *A. macleodii* EP were inoculated into the exudates, and growth was monitored for 15 days. (**C**) Growth curves of bacterial cells in each exudate. Colors indicate bacterial monocultures in exudates obtained from axenic cultures (blue) or co-cultures (magenta), with lines indicating the mean of two biological replicates. Facets indicate the day of culture when the exudate was collected, and the x-axis shows the time spent in that exudate. ANOVA *P*-values from linear models were calculated to assess the effects of the exudate treatment. * indicates that the replicate had a significant effect.

To determine whether *T. pseudonana* differentially released compounds over its growth cycle that either supported or suppressed *A. macleodii* EP growth, we collected cell-free exudates from axenic monocultures of *T. pseudonana* in either mid-exponential or stationary phase. These exudates were then used as growth media for *A. macleodii* EP. Final *A. macleodii* EP cell abundances were 2.4-fold higher when grown in exudates collected from stationary-phase axenic diatoms than from mid-exponential-phase axenic diatoms, consistent with the assumption that organic matter released by *T. pseudonana* into the growth media supported the growth of *A. macleodii* EP (Fig. 2A). We compared the final *A. macleodii* EP cell abundances reached in the two exudate media with those reached in co-cultures, with or without supplementation of organic matter. Final *A. macleodii* EP cell abundances attained on exudates from the exponential-phase axenic diatoms were higher than the co-cultures initiated with mid-exponential-phase *T. pseudonana* cells and lower than the co-cultures supplemented with organic matter (Fig. 2A, left panel). In contrast, final *A. macleodii* EP cell abundances attained on exudates from axenic stationary-phase diatoms were higher than the co-cultures initiated with the stationary-phase diatoms, regardless of whether or not the co-culture media was supplemented with organic matter (Fig. 2A, right panel). These results suggest that *A. macleodii* EP growth was limited in co-culture with either exponential- or stationary-phase diatoms.

We conducted a series of time course experiments to determine whether the apparent defensive response of *T. pseudonana* was a constitutive trait of axenic diatoms or whether it was triggered in response to the presence of A. macleodii EP in co-cultures. We collected exudates every 24 h for 5 days from either axenic exponential *T. pseudonana* cultures or co-cultures of *T. pseudonana* and *A. macleodii* EP. These cell-free exudates were used as media for *A. macleodii* EP mono-cultures, and bacterial growth was monitored for 15 days (Fig. 2B; Fig. S3A). We reasoned that, provided sufficient organic matter was present to support bacterial growth, *A. macleodii* EP would reach higher abundances in exudates from *T. pseudonana* mono-cultures than in exudates from co-cultures, if the bacterial presence triggered a defensive response in *T. pseudonana*. There were no significant differences in *A. macleodii* EP abundances between cells grown in exudates collected immediately after the start of the co-cultures and those collected from axenic diatom mono-cultures (Fig. 2C, facet D0, Table S5). After 24 h, bacterial abundances were higher in mono-culture exudates compared to co-culture-derived exudates (Fig. 2C, facet D1, Table S5). Similar trends were observed at later time points (Fig. 2C, facets D2 and D4, Table S5). Notably, at day 3 (facet D3), variation between replicates was significant, indicating that differences at this time point should be interpreted in the context of replicate-specific effects (Table S5). The rate of drawdown of inorganic nutrients was comparable between the diatom mono-cultures and co-cultures, indicating that *T. pseudonana* grew similarly under both conditions (Fig. S3B). Accumulation of DOC was greater in the mono-culture than in the co-culture, presumably because *A. macleodii* EP drew down DOC for growth. The continued accumulation of DOC in the co-culture, however, suggested that *A. macleodii* EP was unable to take up the DOC at the same rate as the rate of DOC production by *T. pseudonana*. Furthermore, the maximal cell abundance attained by *A. macleodii* EP was significantly higher when the bacterium was grown on the mono-culture exudate than on the co-culture exudate, suggesting that the bacterium was unable to utilize available DOC and attained lower cell numbers on the co-culture exudate (Fig. 2C; Fig. S3C). There was no significant correlation between the ratio of maximum bacterial abundance in mono-cultures and co-cultures and the ratio of organic matter under the two conditions (*R* = 0.3, *P* = 0.47) (Fig. S3D). Thus, the defensive response is triggered by the presence of the bacterium and remains in the liquid phase of the culture after cell filtration, suggesting either the secretion of a defensive compound or changes in culture conditions as potential mechanisms.

Together, these observations suggested that this interaction was governed by a bacterial algicidal effect, the diatom growth phase, the availability of organic matter, and a diatom-triggered defensive response. During the *T. pseudonana* stationary phase, organic matter secretion enabled *A. macleodii* EP to reach cell numbers sufficient to cause diatom death. In contrast, during the exponential phase, *T. pseudonana* appeared to limit bacterial growth through the secretion of defensive compounds, despite adequate availability of organic matter. Addition of organic matter to the co-culture media bypassed the diatom defensive effect, allowing bacterial proliferation and algicidal effects.

### Duo RNA-seq reveals the gene expression regulatory landscape of the *A. macleodii* EP–*T. pseudonana* interaction

**Fig 3:**
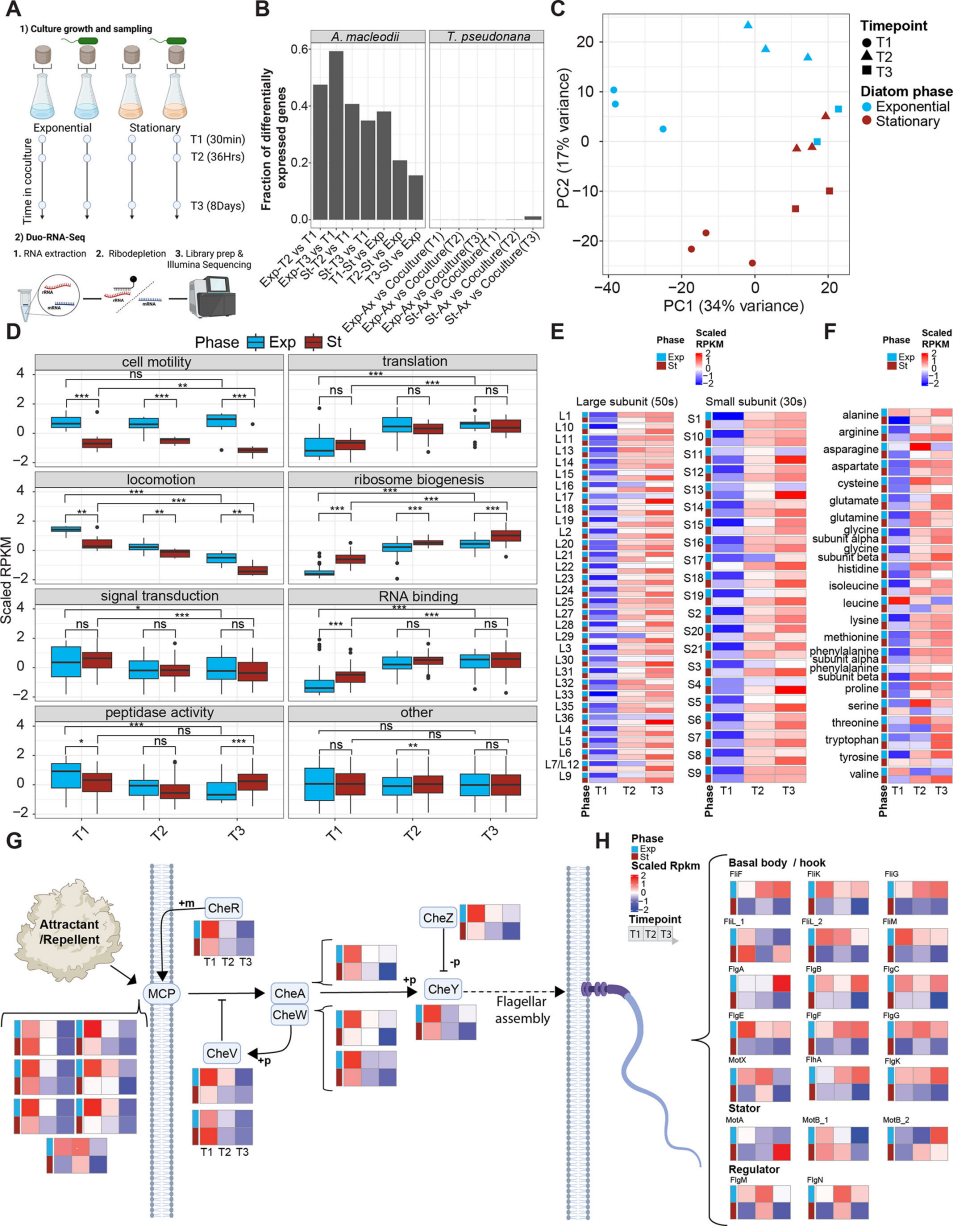
Gene expression regulatory landscape in the *A. macleodii* EP–*T*. *pseudonana* interaction. (**A**) Schematic representation of the sampling design and Duo-RNAseq library preparation. Three biological replicates were taken per experiment; however, for the stationary co-culture (St) and exponential co-culture (Exp), library preparation for replicate 3 was unsuccessful due to low RNA concentration. (**B**) Bar plot showing the fraction of differentially expressed genes in *T. pseudonana* (Thaps3, FilteredModels2) and *A. macleodii* Te101 genomes for specific comparisons. (**C**) Principal component analysis (PCA) using scaled reads per kilobase per million mapped reads (RPKM) from *A. macleodii* EP. (**D**) Box plot of scaled RPKM values for selected GO groups in *A. macleodii* EP across time points and between *T. pseudonana* initial growth phases: exponential (blue) and stationary (magenta). RPKM values are averaged across triplicates. Statistical significance was assessed using Wilcoxon rank-sum tests for T1 vs T3 within each condition and for St vs Exp at each time point, with significance indicated as *P < 0.05, **P < 0.01, ***P < 0.001, and ns (not significant). (**E**) Heatmap showing scaled RPKM values for ribosomal proteins identified using the KEGG BLAST KOALA tool and assigned to the Ribosome KEGG pathway (ko031011). (**F**) Similar to panel **E**, but for aminoacyl-tRNA synthetases from the aminoacyl-tRNA biosynthesis KEGG pathway (ko00970). (**G**) Schematic representation of gene expression changes in the Bacterial Chemotaxis (ko02030) KEGG pathway. Genes belonging to the GO category *locomotion* fall within this pathway. Heatmaps were generated from the scaled average RPKM values between replicates. Row annotations distinguish between exponential (blue) and stationary (magenta) samples, while each column represents a time point (T1, T2, T3), organized from left to right. Gene names are indicated above each heatmap. The dashed arrow illustrates how chemotaxis signaling, mediated by methyl-accepting chemotaxis proteins (MCPs), controls flagellar assembly in response to environmental signals. The "attractant/repellent blob" represents the chemical gradients sensed by MCPs, which modulate bacterial movement. MCPs are located in the cell membrane, where they interact with chemical signals, while the flagella, also in the membrane, are responsible for bacterial swimming. (**H**) As shown in panel **G**, but for the Flagellar Assembly (ko02040) KEGG pathway. Genes were divided into the categories: basal body/hook, stator, and regulator. Genes belonging to the GO category cell motility fall within this pathway.

To investigate the dynamics of gene expression in *A. macleodii* EP and *T. pseudonana* co-culture, we measured transcriptional profiles at 30 min, 36 h, and 8 days for co-cultures initiated with either exponential-phase or stationary-phase diatoms, as well as for an axenic diatom mono-culture (Fig. 3A; Fig. S4A). We aligned the resulting sequence reads to the *T. pseudonana* genome and to 14 *A. macleodii* genomes. The greatest number of reads aligned to the *T. pseudonana* and the *A. macleodii* Te101 genomes (25) (Fig. S4D).

To explore the differences triggered by bacterial presence and the effects of diatom growth phase and time in co-culture, we identified differentially expressed genes in the corresponding pairwise comparisons (Fig. 3B; Fig. S5A). PCA indicated that diatom transcript abundance profiles changed as exponential-phase cultures progressed through the growth phases in both axenic mono-cultures and co-cultures, impacting the transcript levels of ~3,000 genes (~40%). In contrast, the presence of bacteria elicited a modest transcriptional response in stationary-phase diatoms, with ~90 genes (~0.7%) of *T. pseudonana* differentially expressed (Fig. S5B). This suggests that the presence of the bacterium did not trigger a transcriptome-wide response in the diatom. Among the genes differentially expressed in *T. pseudonana* in response to the presence of *A. macleodii* EP, we detected three transcripts with predicted serine protease inhibitor activity: THAPSDRAFT_6004, THAPSDRAFT_36303a, and THAPSDRAFT_24702. These gene products could potentially contribute to limiting bacterial pathogenicity and their ability to extract resources from the diatom by truncating the function of bacterial proteases (26) (Table S2). We detected a natriuretic peptide receptor (THAPSDRAFT_263505), predicted to be located in the membrane, and a peripheral-type benzodiazepine receptor (THAPSDRAFT_2880), predicted to be located in the mitochondria; both were differentially expressed and could potentially be associated in triggering a signaling cascade induced by the bacterium (27). Additionally, a serine/threonine protein kinase (THAPSDRAFT_1388) and a serine/threonine protein phosphatase (THAPSDRAFT_36303) were differentially expressed (Table S2). We also observed a significant fraction of differentially expressed genes associated with chromatin structure and dynamics KOG class (Table S2; Fig. S5C). Ten histones were downregulated in co-culture with the bacterium, suggesting chromatin reconfiguration (Table S2; Fig. S5C).

In contrast, 20 to 40% of *A. macleodii* EP genes (200–600 genes) were differentially expressed during co-culture, depending on the sampling time point (Fig. 3B; Fig. S5A). PCA-based analyses indicated that ~34% of the variability was described by the first principal component (PC1), which followed the progression of time in co-culture, while ~16% of the variability was described by PC2, which separated bacteria co-cultured with *T. pseudonana* in exponential versus stationary phase (Fig. 3C). The most significant differences occurred at 30 min, suggesting that the bacterium rapidly sensed and differentially responded to the conditions created by either exponential-phase or stationary-phase diatoms. Overall, these results suggest that the progression of the diatom culture and the initial conditions of the co-culture shape both the outcome of the interaction and the transcriptome of *A. macleodii* EP.

A Gene Ontology (GO) enrichment analysis of differentially expressed *A. macleodii* EP genes revealed consistent changes across time when the co-cultures were initiated with either stationary-phase or exponential-phase diatoms (Fig. S5D). At 30 min, upregulated *A. macleodii* EP genes were enriched for Biological Process GO terms related to cell motility, locomotion, and signal transduction, as well as Molecular Function GO terms associated with peptidase activity. At the 36-hour and 8-day time points, enriched Biological Process GO terms included translation and ribosome biogenesis, while Molecular Function terms were related to RNA binding. The genes categorized with the GO terms “locomotion” and “signal transduction” were downregulated over time in bacteria co-cultured either with exponential-phase or stationary-phase diatoms (Fig. 3D). Conversely, the genes categorized with the GO terms “ribosome biogenesis,” “translation,” and “RNA binding” were upregulated over time in bacteria co-cultured either with exponential-phase or stationary-phase *T. pseudonana* (Fig. 3D). Moreover, fold-change differences across GO groups were significantly higher at 30 min (T1) after *A. macleodii* EP was added to either co-culture, except for cell motility, suggesting a rapid transcriptional adjustment to environmental conditions (Fig. S5E). These dynamics were not observed in the distribution of other GO groups, highlighting the specificity of the response (Fig. 3D). Transcriptional profiles appear quantitative, with transcript abundances shifting progressively over time from low to high (or vice versa). Together, these patterns reflect a gradual transition from a mobile- and sensing-oriented state toward a growth-oriented state, driven by adaptation to diatom-derived substrates.

The progression of gene expression patterns for genes with predicted peptidase activity and cell motility differed between co-cultures initiated with exponential- or stationary-phase diatoms. Co-cultures initiated with stationary-phase *T. pseudonana* showed consistent downregulation of motility genes, whereas exponential-phase–initiated co-cultures, even after reaching stationary phase at day 8, never reached the same levels, highlighting the lasting influence of the diatoms’ initial growth state on bacterial swimming (Fig. 3D). In exponential-phase co-cultures, transcript levels for the peptidase-encoding genes decreased consistently across all time points, whereas in stationary-phase co-cultures, transcript levels initially decreased but recovered by 8 days (T3) to the levels observed at 0.5 h (T1). Additionally, at 0.5 h (T1), expression was significantly higher in the bacterium co-cultured with exponential-phase rather than stationary-phase diatoms; the relationship was reversed at 8 days (T3) (Fig. 3D). Furthermore, peptidase-predicted gene transcripts levels changed not only in magnitude over time but also in composition (Fig. S6A, left). Similar changes across time and stages in transcript levels were detectable for genes encoding *A. macleodii* EP proteases (Fig. S6A, right). These results demonstrate that both the quantity and identity of transcripts associated with peptidases and proteases are influenced by time in co-culture, the physiological state of the diatoms, and the progression of the interaction.

Three main categories of differentially transcribed genes capture the transition in bacterial functions over time and in response to the co-culture environment. The first category comprises genes encoding tRNA synthetases as well as large and small subunit ribosomal proteins. Transcript levels for these genes increased through time in all co-cultures and were consistently lower when cultures were initiated with exponential-phase diatoms, especially at the 0.5 h (T1) time point (Fig. 3E). Similarly, transcripts for 18 of the 22 detectable aminoacyl-tRNA synthetases increased over time, and 15 of the 22 exhibited higher abundances at T1 when co-cultured with stationary-phase diatoms (Fig. 3F). Ten remained elevated at 8 days (T3) when stationary-phase diatoms were used for inoculation. A second category includes chemotaxis signaling genes, which were downregulated over time regardless of the diatoms’ initial growth phase (Fig. 3G). This gene set includes methyl-accepting chemotaxis proteins (MCPs), which serve as membrane-bound sensors of chemical gradients, as well as proteins involved in downstream signal transduction, such as CheA; motor regulators CheY and CheZ; adaptation proteins including CheR; and coupling proteins CheV and CheW. A third category comprises flagellar genes, whose expression patterns diverged sharply between exponential- and stationary-phase conditions (Fig. 3H). Genes encoding the basal body and hook structures were significantly upregulated when *A. macleodii* EP was co-cultured with exponential-phase diatoms, but downregulated with stationary-phase diatoms. Genes encoding components of the stator complex (e.g., MotA, MotB) and regulators such as FlgM and FlgN displayed gene-specific responses: one MotB paralog decreased over time while the other increased, MotA remained stable, and FlgM/FlgN showed no clear dependence on the initial diatom phase. These findings indicate that *A. macleodii* EP modulates the balance between motility and growth during co-culture, depending on the diatom’s growth phase and time in interaction. This shift from searching to exploiting a nutrient source is accompanied by dynamic, phase-dependent changes in peptidase and protease expression (Fig. 3D; Fig. S6A), reflecting coordinated adjustments to the diatoms’ physiological state.

### *A. macleodii* EP algicidal effect is mediated via the secretion of an algicidal compound

**Fig 4:**
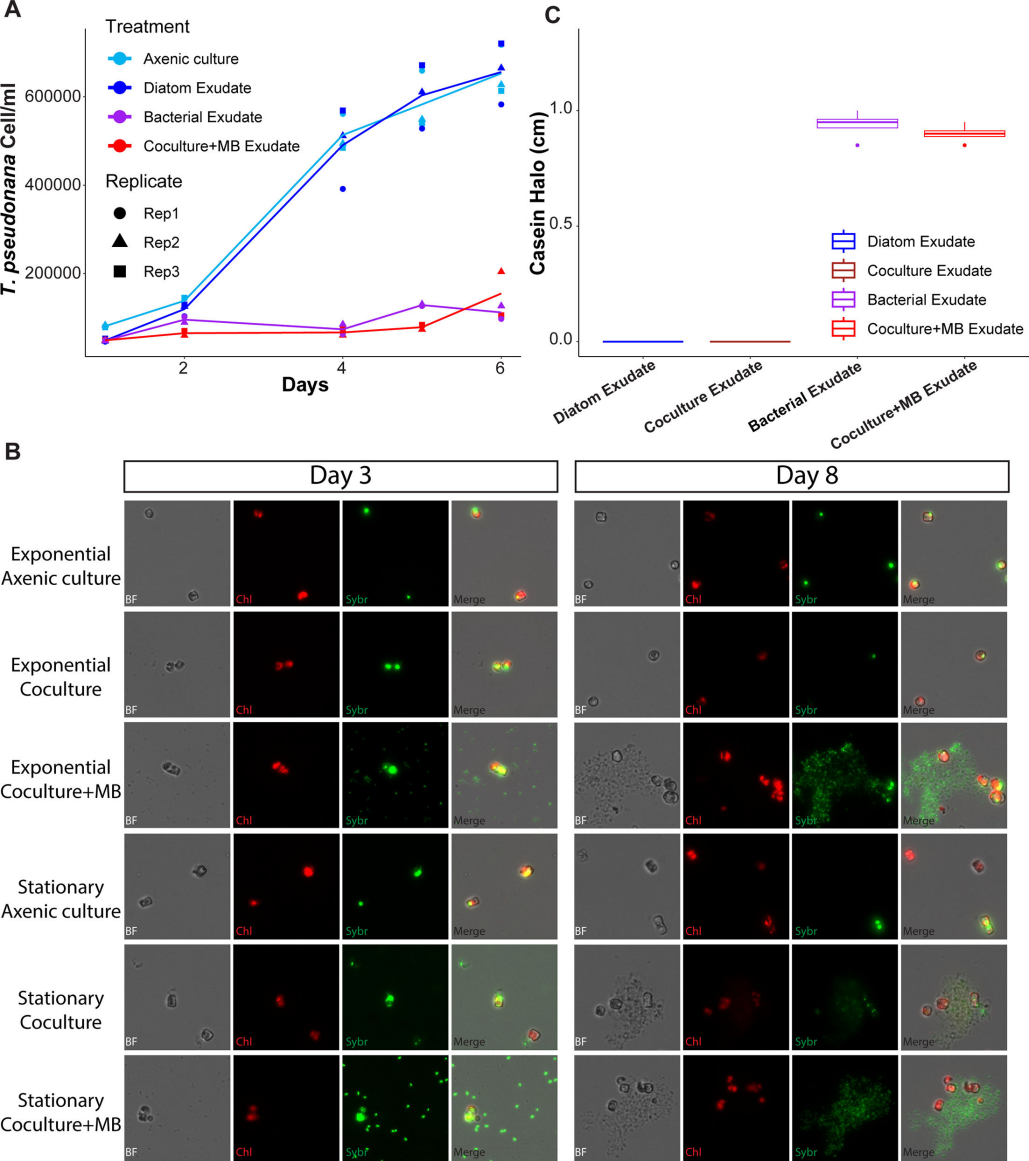
Bacterial–diatom interaction occurs in two stages, likely mediated by the secretion of peptidases that cause cell lysis. (**A**) Scatter plot of diatom cell counts measured by flow cytometry, with lines representing the average of three replicates. Colors distinguish different media treatments. (**B**) Fluorescence microscopy images showing the location of diatom and bacterial cells under specified conditions and times. Images include brightfield, red chlorophyll autofluorescence, green SYBR Green-stained DNA, and a merged view of all channels. (**C**) Box plot of the halo diameter generated on casein plates by different filtrates, using data from two biological replicates with two technical replicas each.

The transcriptional data indicate that *A. macleodii* EP transitioned from a chemotactic swimming behavior during the early phase of co-culture with exponential- and stationary-phase diatoms, when bacterial abundance was low, to a growth-associated behavior in later co-culture phases, each characterized by the expression of a specific set of proteases and peptidases. To determine whether a secreted compound induced (or mediated) the algicidal effect, and whether the presence of *T. pseudonana* was required to trigger the secretion of such compounds, we attempted to grow *T. pseudonana* in exudates collected from (i) axenic diatom mono-cultures, (ii) *A. macleodii* EP mono-cultures grown in diatom media, and (iii) co-cultures initiated with exponential-phase diatoms, supplemented with MB. As expected, the growth rate of *T. pseudonana* in its axenic culture exudate was not significantly different from growth in axenic culture media (Fig. 4A). In contrast, *T. pseudonana* did not grow in either the *A. macleodii* EP axenic mono-culture exudate supplemented with MB or the co-culture exudate supplemented with MB (ANOVA followed by post hoc pairwise comparisons with Bonferroni-adjusted *P*-values: axenic culture–diatom exudate = 1, axenic culture–bacterial exudate < 0.0001, axenic culture–co-culture + MB exudate < 0.0001, bacterial exudate–co-culture + MB exudate = 1) (Fig. 4A). These results implied that (i) previous exposure to diatoms is not necessary to elicit the algicidal effect in *A. macleodii* EP and that (ii) the algicidal agent is secreted into the media by the bacterium.

Next, we examined the phenotypic behavior of *A. macleodii* EP over time in co-cultures initiated with either exponential- or stationary-phase *T. pseudonana*, with or without MB supplementation. We hypothesized that, given that exudates were sufficient for algicidal activity, *A. macleodii* EP should not cluster around *T. pseudonana* cells in these co-cultures. After 3 days in co-culture, at peak algicidal activity, *A. macleodii* EP cells were detectable by microscopy in both the stationary-phase co-cultures and in those supplemented with MB, but not in the exponential-phase co-cultures (Fig. 1A and B). At this early time point, the bacterial cells appeared randomly distributed, with no clear association with *T. pseudonana* cells (Fig. 4B, left). By day 8, cultures initiated with stationary-phase diatoms or supplemented with MB—where diatom cell numbers were significantly reduced—exhibited a shift in distribution pattern, with *A. macleodii* EP cells clustering around apparent diatom debris. (Fig. 4B, right). The ability of Alteromonas species to form aggregates through the secretion of sticky exopolymers has been demonstrated (28), and at least one gene required for exopolymer production, *UDP-glucose 4-epimerase*, is conserved across *A. macleodii* strains (29). We observed increased aggregate formation at the 8-day time point, coincident with a significant accumulation of *UDP-glucose 4-epimerase* transcripts over time (Fig. S7). Notably, transcript levels did not significantly differ between stationary- and exponential-phase co-cultures, suggesting that the differences in absolute aggregation are likely driven by higher cell densities in co-culture with stationary-phase diatoms, rather than by differences in gene expression *per se* (Fig. S7). This spatial reorganization suggested that the bacterium was actively interacting with the lysed diatom material, potentially breaking it down for necessary substrates.

Motivated by the observation that peptidases changed their expression over the course of the experiment (Fig. 3D; Fig. S6A) and that the PSORTb algorithm (30) predicted that approximately 50% of the peptidases were extracellular (Fig. S6B), we used casein plates to test whether active peptidases or proteases were secreted into the different cell-free exudates. The presence of an active peptidase or protease within the exudate will cause the white color of the plate to fade, generating a measurable halo. Cell-free exudates from the *A. macleodii* EP mono-culture or exponential co-cultures supplemented with MB both prevented diatom growth and generated casein halos, indicating the presence of active proteases or peptidases (Fig. 4C; Fig. S6C). The cell-free exudates from exponential co-cultures without the MB supplement did not generate a halo, despite the bacteria having the highest transcript levels associated with peptidases (Fig. 4C; Fig. S6C). Presumably, this is a result of the low cell concentrations of *A. macleodii* EP in unsupplemented co-cultures, likely due to limited availability of organic matter and the diatom defensive response (Fig. 3D and 4C).

### *A. macleodii* growth-motility trade-off is a conserved regulatory feature, but its directional progression in co-culture is host-specific

To determine whether *A. macleodii* EP displayed similar transcriptional patterns regardless of the co-culture host, we reanalyzed publicly available RNA-seq data from a co-culture experiment of *A. macleodii* MIT1002 with the marine photosynthetic bacterium *Prochlorococcus* NATL2A (31). The MIT1002 reads were aligned to the Te101 genome (25) to allow a comparison of genes between the two experiments.

**Fig 5:**
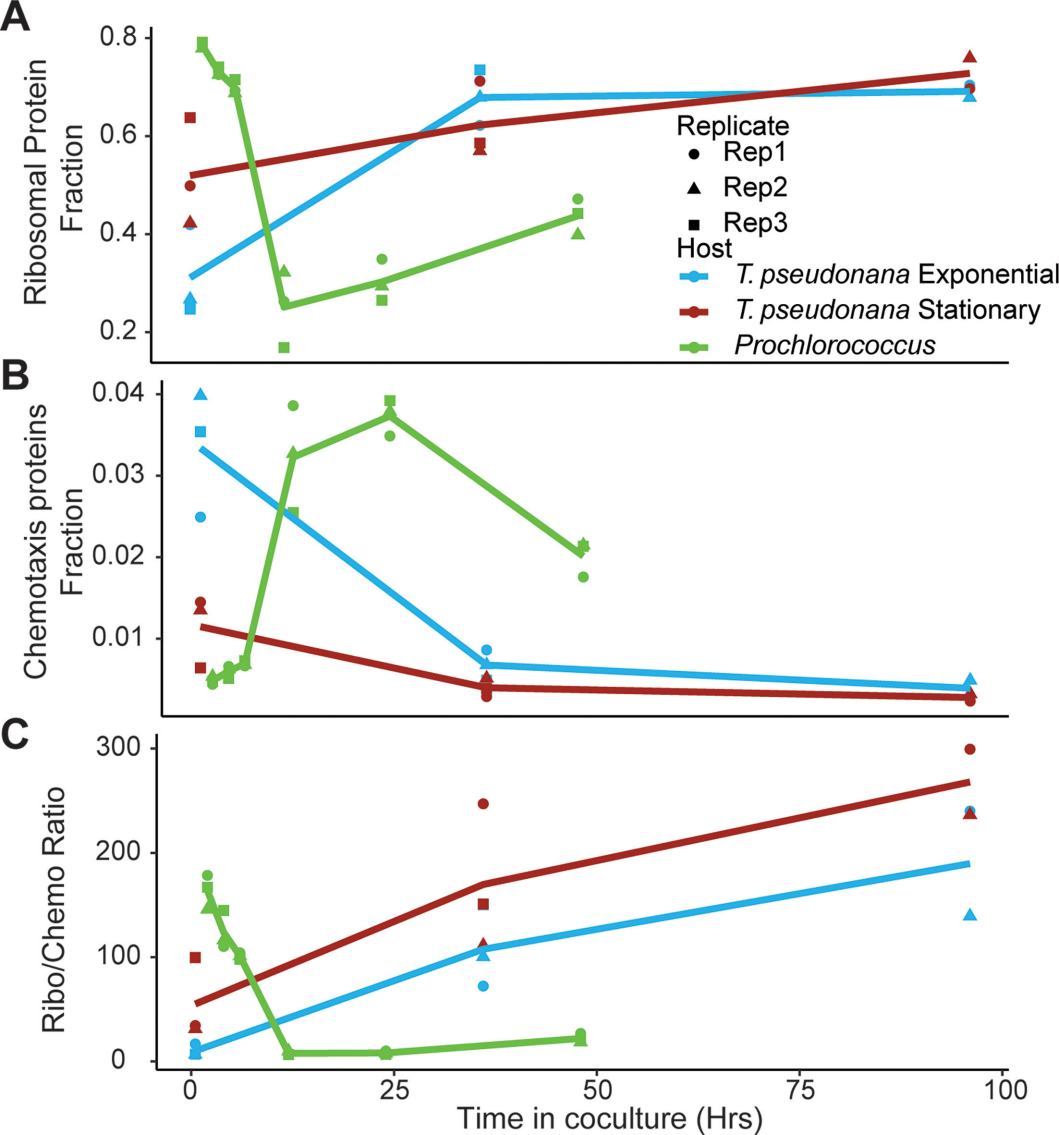
*A. macleodii* expression progression in co-culture is host-dependent, fluctuating between motility and growth. Scatter plots showing ribosomal proteins (**A**), chemotaxis-related genes (**B**), and the ratio between them (**C**), representing their fraction of transcription in co-culture with *T. pseudonana* (exponential, blue; stationary, magenta) and *Prochlorococcus* (green). Replicates are indicated by different symbols (circles, triangles, squares), with lines representing the average of three biological replicates (except for T3 *T. pseudonana* co-cultures, which were run in duplicate).

The *A. macleodii* EP transcript-level profile in co-culture with *T. pseudonana* displayed an opposite pattern to that of *A. macleodii* MIT1002 in co-culture with *Prochlorococcus* NATL2A: genes transcribed at high levels at the 30-min time point when co-cultured with *T. pseudonana* were transcribed at high levels at the 12-, 24-, and 48-hour time points when co-cultured with *Prochlorococcus*, and vice versa (Fig. S8). To further characterize this pattern, we focused on those genes encoding ribosomal proteins and chemotaxis-related proteins (Fig. 3E). The fraction of transcripts encoding ribosomal proteins increased over time in co-culture with *T. pseudonana,* whereas this fraction decreased over time in co-culture with *Prochlorococcus* (Fig. 5A). Genes encoding chemotaxis proteins displayed the opposite pattern: the fraction of transcripts encoding chemotaxis proteins decreased over time in co-culture with *T. pseudonana,* whereas this fraction increased over time in co-culture with *Prochlorococcus* (Fig. 5B). Finally, we calculated the ratio between the fractions of transcripts encoding ribosomal proteins and chemotaxis-related proteins as a proxy for cell growth or motility. Based on this proxy, *A. macleodii* EP shifts from a motility to a growth profile in co-culture with *T. pseudonana*, whereas *A. macleodii* MIT1002 shifts from a growth to a motility profile in co-culture with *Prochlorococcus* (Fig. 5C). We conclude that the progression of gene expression in co-cultures is host-dependent and potentially strain-dependent, and that *A. macleodii* adjusts the growth-to-motility ratio in response to its environment.

Finally, to dissect whether the observed changes are driven by the host or due to changes in substrate concentrations, we reanalyzed monocultures transcriptomic data from Mank et al. (32), in which *Alteromonas macleodii* ATCC 27126 was subjected to iron and carbon limitation. We aligned reads from this experiment to the Te101 genome for direct comparison with our co-culture experiments. Overall, our analysis indicates that proteases, peptidases, ribosomal proteins, and most chemotaxis genes are conserved across EP, ATCC 27126, and MIT1002, despite minor differences such as additional proteases in EP and ATCC 27126, five missing peptidases in MIT1002, and the loss of mostly redundant chemotaxis gene copies, indicating that core protein machineries are broadly preserved (Fig. S9).

Bacterial monoculture transcriptomes clustered separately from co-cultures with diatoms, regardless of treatment or time point (Fig. S10A), reflecting distinct transcriptional profiles under the different growth conditions. Fold-change correlation analyses compared gene-by-gene expression changes under carbon and iron limitation (relative to replete monocultures) with those observed during co-culture. Transcriptome fold changes under carbon limitation showed a moderate correlation (~0.4, *P* < 0.05) with co-culture expression shifts, both across time points (T3/T1) and between diatom growth phases at T1. This suggests that approximately 20% of the variance in co-culture transcriptional patterns can be explained by carbon-driven responses (Fig. S10A). In contrast, transcriptional profiles associated with iron limitation showed little correspondence with the co-culture patterns (Fig. S10B). Ribosomal protein fractions declined under both carbon and iron limitation, consistent with reduced growth, while chemotaxis proteins responded selectively to carbon limitation (Fig. S10C), reflecting dependence on diatom-derived carbon and a transition from motility to growth. In contrast, temporal changes in proteases and peptidases were observed only in co-cultures (Fig. S10D), indicating that these responses are specifically induced by diatom interactions rather than by changes in bulk carbon availability alone.

## DISCUSSION

**Fig 6:**
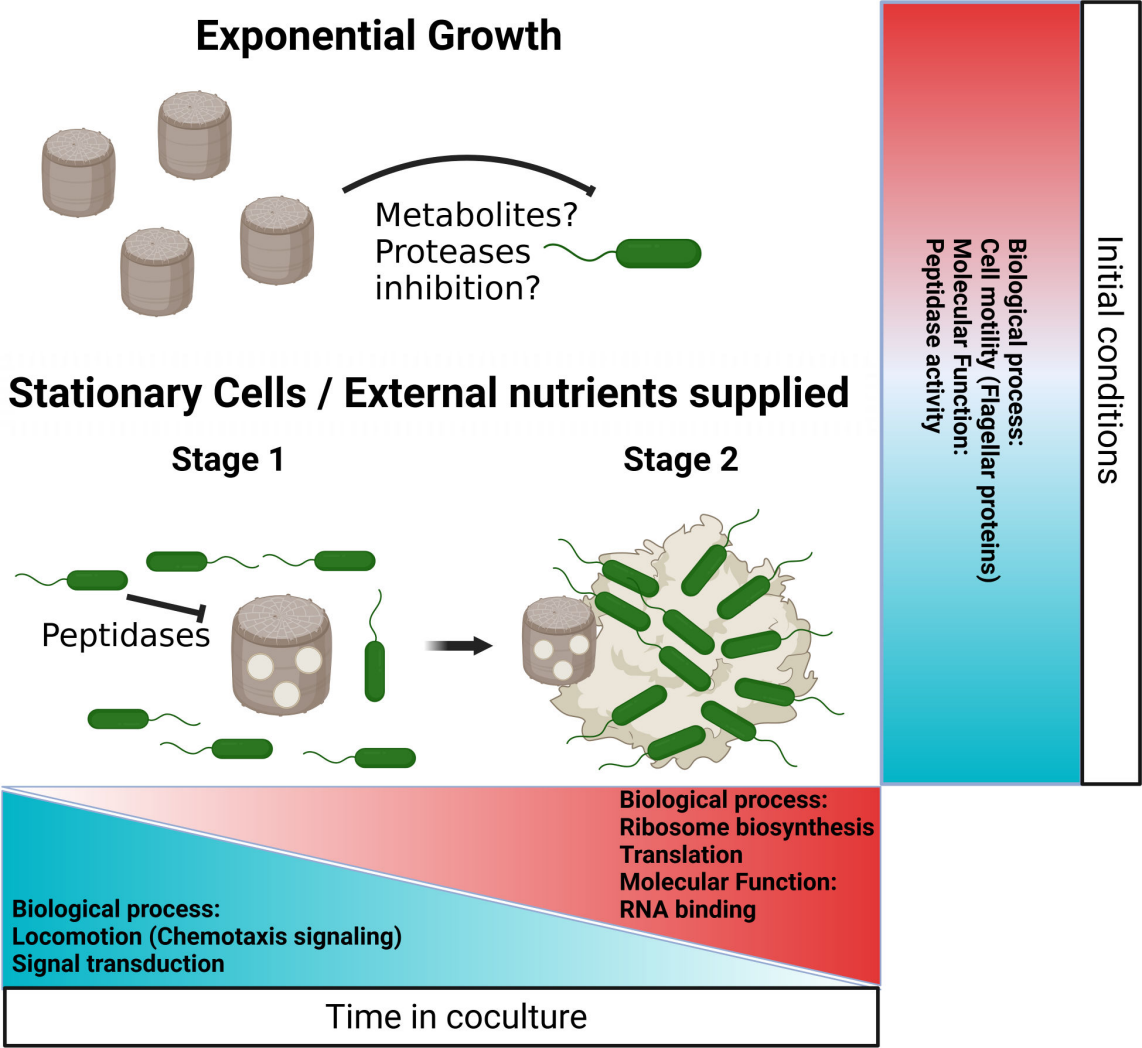
Model schematic of *A. macleodii* EP and *T. pseudonana* interactions. The initial conditions of *T. pseudonana* culture determine the outcome of its interaction with *A. macleodii* EP. During exponential growth, the diatom appears to control bacterial proliferation; however, in the stationary phase, or if external nutrients are supplied, bacterial cells thrive and exhibit an algicidal effect. This effect unfolds in two stages: first, bacterial growth accompanied by the secretion of a cocktail of peptidases, and subsequently, the formation of cell clusters around diatom debris. In response, *A. macleodii* EP senses its environment and adjusts the expression of genes involved in key biological functions to suit the prevailing conditions. Chemotaxis signaling, ribosomal proteins, and translation-related genes shift progressively through time in response to substrate availability, reflecting dynamic adaptation. In contrast, peptidases and flagellar gene expression are determined by the diatom’s initial growth phase, showing long-lasting effects of starting conditions.

In this study, we explored how diatom growth phase physiology influences interactions with bacteria. We identified a dynamic interaction between a recently isolated Equatorial Pacific strain of *A. macleodii* and the model diatom *T. pseudonana*. When grown as co-cultures, *A. macleodii* EP grew when co-cultures were initiated with *T. pseudonana* in stationary phase or when an external substrate source was provided to co-cultures initiated with either stationary- or exponential-phase *T. pseudonana*. We propose a two-stage model of pathogenic progression: initially, *A. macleodii* EP releases peptidases and/or proteases that compromise diatom cell structures, leading to cell lysis; subsequently, bacterial clusters accumulate around the resulting diatom debris. Moreover, we show that *A. macleodii* EP growth is restricted in co-culture with *T. pseudonana*, as the bacteria fail to reach the abundances observed in axenic diatom exudates. This indicates that the diatom actively limits bacterial proliferation, likely through the secretion of defensive compounds triggered by the presence of *A. macleodii* EP (Fig. 6). Importantly, some experiments were performed using only two replicates, limiting statistical inference of the study. Nevertheless, this simplified artificial model provides a useful system to study host phase effects in bacterial–phytoplankton interactions, despite not fully capturing natural environmental conditions.

Our study both overlaps with and differs from previous work on the co-culture of *T. pseudonana* with the L15 strain of *A. macleodii* (7). Multiple differences are apparent between the response of *T. pseudonana* to the L15 and EP strains of *A. macleodii*. First, L15 grew in co-culture with exponentially growing *T. pseudonana*, shifting from mutualism to weak parasitism, whereas EP did not grow with exponential *T. pseudonana* cells under similar conditions. This suggests that L15 is somehow unaffected by the putative diatom defensive response, either because the response is not triggered by L15 or because the defensive response is ineffective against L15. Second, the algicidal activity of L15 appears to be inducible by the addition of organic matter (Zobell media), whereas the algicidal activity of EP appears constitutive, with the addition of MB to the co-culture with EP overcoming the defensive response of *T. pseudonana*. Third, L15 swimming velocity increased upon exposure to stationary-phase diatom exudate, suggesting enhanced chemotaxis, whereas EP displayed reduced transcript levels of chemotaxis-related genes when co-cultured with stationary-phase diatoms, suggesting reduced chemotaxis. This response of EP is similar to ATCC 27126 under carbon-replete conditions (Fig. S10C) (32). Finally, the algicidal behavior of both L15 and EP relies on proteases. However, L15 appears to require cell attachment (7), whereas the EP exudates alone are sufficient to induce algicidal activity.

The different behaviors of the two *A. macleodii* strains may reflect different adaptations to their habitats: L15 was isolated from a coastal bloom, whereas EP was isolated from the open ocean. Coastal environments support higher diatom biomass, particularly during blooms. The ability of L15 to enhance swimming speeds and attach to stationary-phase diatoms may reflect T15 algicidal behavior at the end of diatom blooms when the diatoms tend to aggregate (33, 34). In contrast, EP increases swimming when resources are limited, suggesting an open-ocean strategy in which enhanced motility helps it locate potential targets and secrete algicidal compounds that kill the cells it encounters. Co-cultures of additional *A. macleodii* strains with algae have shown competition for nitrate when co-cultured with the pennate diatom *Phaeodactylum tricornutum* (16), and a mutualistic interaction with the haptophyte *Isochrysis galbana* (14). Together, these results underscore the complexity and context-dependence of bacteria-algal interactions, highlighting how host physiology and environmental factors jointly shape microbial relationships in marine ecosystems.

Across *T. pseudonana* growth phases, *A. macleodii* EP shifts its transcriptome balancing chemotaxis and growth in response to the diatom’s state and substrate availability (Fig. 6). This pattern is consistent with a previously proposed trade-off between bacterial motility and growth, wherein the energetic and proteomic costs of maintaining motility machinery constrain investment in biosynthetic processes (35, 36). This energetic and proteomic cost can divert resources away from growth, especially in environments where motility offers little benefit, such as well-mixed or nutrient-rich conditions (35, 36). Previous studies in *E. coli* and other organisms have shown that translation is a limiting factor for growth (37–39), and the fraction of proteins allocated to the ribosome reflects the growth capacity. In our system, the chemotactic sensory system, ribosomal proteins, and other translation-related transcripts abundance change progressively over time in co-culture. These changes may reflect an indirect response to changes in organic carbon availability as the diatom progresses through its growth phases. In contrast, the expression of peptidases and flagellar genes was determined by the initial growth phase of the diatom in the co-culture. These responses suggest that part of the transcriptional program follows general metabolic and substrate dynamics, while another component reflects a potential diatom-specific effect, revealing the long-lasting impact of the diatom’s growth phase at the start of co-culture on bacterial responses and, consequently, on the dynamics of the interaction (Fig. 6).

*A. macleodii* strains are flexible in how they interact with other microorganisms, with interactions ranging from pathogenic to mutualistic. Several strains of *A. macleodii* have been studied, and differences in their ecology have been inferred from their genomes and substrate utilization patterns (29, 40). By comparing the co-culture gene expression profiles from this study to previous experiments with *Prochlorococcus* (31), we found that *A. macleodii* EP shifts from a motile to a growth expression profile in co-culture with *T. pseudonana*, while *A. macleodii* MIT1002 follows the opposite trajectory in co-culture with *Prochlorococcus*. We cannot rule out the possibility that these differences are due to genetic content variations between the two bacterial strains. Moreover, the MIT1002–*Prochlorococcus* system represents a long-term natural co-existence, whereas the EP–*T*. *pseudonana* pairing is a laboratory model. The alternative explanation is that the observed differences result from the different nature of the two interactions. In co-culture with *Prochlorococcus*, *A. macleodii* MIT1002 engages in a mutualistic relationship (31), whereas in co-culture with *T. pseudonana*, *A. macleodii* EP displays a pathogenic behavior. Moreover, the presence of *A. macleodii* MIT1002 facilitates *Prochlorococcus* survival in the dark by exchanging essential compounds, showing how the two species support each other’s metabolism (41, 42). Additional studies have highlighted the collaborative role of *A. macleodii* in the ocean, showing that it secretes siderophores that mobilize iron and extracellular enzymes to break down complex polysaccharides for community use (43, 44). We hypothesize that the balance between motility and growth in *A. macleodii* is determined indirectly, in a host-dependent manner, by changes in carbon availability, which act as a key environmental cue modulating the outcome of microbial interactions. It is noteworthy that most EP reads mapped to Te101, a strain originally isolated from a *Trichodesmium* co-culture (25), suggesting that the genomic and ecological traits enabling *A. macleodii* to interact with diverse phytoplankton may be broadly conserved across strains that encounter different hosts in the marine environment.

Our finding that the interactions of *A. macleodii* with phytoplankton vary depending on both the hosts and their growth phase has wide-ranging ecological implications, such as potential roles in regulating algal blooms, reshaping microbial community composition, and enhancing carbon export through aggregate formation (45). Laboratory experiments with *Thalassiosira rotula* and *Skeletonema costatum* demonstrated that bacterial community composition depends on diatom growth phase (46). Because diatom blooms follow a similar trajectory, with an exponential growth phase followed by decline at peak cell abundance (47), the observed changes in bacterial community composition (24, 48) are likely driven not only by diatom species composition but also by shifts in diatom growth phase. Commonly, *Alteromonadaceae* are present at relatively low abundance during the initial bloom phase and increase in abundance as the bloom progresses, coinciding with diatom aggregation and sinking (49). Our results are consistent with a selective modulation of bacterial growth during diatom bloom development: *T. pseudonana* suppresses *A. macleodii* during the exponential phase, but not other bacteria such as *R. pomeroyi* or *Sulfitobacter* sp*.* (Fig. S2B). This specificity implies that either the inhibitory mechanism is triggered by particular bacterial traits, that some bacteria are naturally resistant, or a combination of both. The coastal/estuarine T. pseudonana is unlikely to co-occur with *A. macleodii* EP, suggesting that the inhibition response we observed may impact a broader group of bacteria, potentially *Alteromonadaceae*. Distinct bacterial genetic profiles are associated with mutualistic (50–52) and antagonistic interactions, suggesting that diatoms sense and respond to the traits of their bacterial partners (53). Diatoms may deploy defensive strategies to limit exploitation by opportunistic copiotrophs such as *Alteromonadaceae*, conserve resources during early growth, and promote the establishment of mutualistic partners. Interactions with other bacteria can also protect algae against algicidal species (11), raising the possibility that community context may modulate these defensive outcomes. Whether such interactions influence the algicidal activity of *A. macleodii* remains unknown. Nevertheless, under high-nutrient conditions, the copiotrophic lifestyle of *A. macleodii* could give it a competitive advantage, potentially allowing it to reach concentrations sufficient to influence bloom dynamics. Additionally, particle aggregation increases as a consequence of algal blooms (54, 55). Consistent with our findings, experiments with *T. rotula* have shown that bacterial colonization and aggregation are more efficient when *T. rotula* cells are in stationary phase (56). The formation of aggregates by *A. macleodii* could affect microbial interactions and carbon cycling: larger aggregates are more likely to be grazed by larger zooplankton, facilitating nutrient transfer up the food web, or their increased size can accelerate sinking rates, potentially enhancing carbon export from the surface to the deep ocean (55).

The molecular mechanism of the observed inhibitory effect of *T. pseudonana* remains to be discovered. One possibility is the secretion of metabolites that limit bacterial growth. Diatoms secrete specific compounds or enzymes that control bacterial companions, and *T. pseudonana* can secrete compounds affecting copepods (13, 57–59). This potential mechanism does not require direct cell-to-cell contact. Analyzing the secreted metabolome of co-cultures, compared to mono-cultures, will help identify potential regulatory metabolites. The limited transcriptional response of *T. pseudonana* to *A. macleodii* EP suggests that the effect is constrained rather than transcriptome-wide, and that major regulatory adjustments may occur at levels other than transcription. There is evidence in other organisms of post-transcriptional (60, 61) and post-translational (62, 63) responses to bacterial presence. Further studies on post-transcriptional regulation may provide insights into the mechanisms underlying *T. pseudonana*’s defensive response.

In conclusion, co-culturing *A. macleodii* EP with *T. pseudonana* revealed that bacterial transcriptional programs and phenotypes are modulated by host growth phase, substrate availability, and potential inducible defense responses. These findings build on prior work linking phytoplankton physiology to microbial community structure and extend it by identifying molecular mechanisms through which the host state regulates bacterial behavior. How growth phase-driven regulatory dynamics influence ecosystem-scale processes such as bloom collapse, particle aggregation, and carbon export in marine environments remains a critical area for further study.

## MATERIALS AND METHODS

### Equatorial Pacific *A. macleodii* isolation and identification

The bacterium *A. macleodii* EP was isolated during the Gradients 4 cruise (TN397) in the equatorial Pacific in December 2022. The bacterium was isolated from seawater collected at Station 9 (140° W, 4.75° N) during a CTD cast at 15 m depth. Approximately 1 mL of collected seawater was spread onto 1/2 YTSS plates (2 g yeast extract, 1.5 g tryptone, 20 g sea salts, 15 g agar in 1 L of distilled water), and the plates were incubated for about 4 days at 20°C. Single colonies were picked and restreaked on 1/2 YTSS media plates until a pure culture was obtained. The identity of *A. macleodii* EP was determined by sequencing the 16S rRNA region (Sanger sequencing at Azenta) (Data S1) and comparing the sequence to publicly available data via NCBI BLAST using the rRNA_typestrains/16S_ribosomal_RNA database. The 16S phylogenetic tree was generated using publicly available 16S sequences from NCBI that had high similarity to *A. macleodii* EP based on BLAST results (Table S1). Multiple alignment was done with MUSCLE (64), the aligned sequences were trimmed to be the same length in Geneious (Geneious Prime 2025.0.3), and the maximum likelihood tree was built using RaxML version 8.2.4 with 1,000 bootstraps (65) (parameters: -m GTRGAMMA -f a -x 1 -N 1000 -p 1). The tree was visualized using Geneious.

### Co-culture experiments

The diatom *T. pseudonana* CCMP 1335 strain was maintained as a semicontinuous batch culture in 30 mL L1 + Si medium (NCMA) 66), which contains natural sea salts supplemented with the macronutrients nitrate (882 µM), phosphate (36 µM), and silicate (106 µM), as well as trace metals and vitamins. Cultures were kept at 20°C under a 16:8 h light-dark cycle with ~160  µmol photons m⁻² s⁻¹. *In vivo* chlorophyll a fluorescence was monitored (Turner Designs 10-AU fluorometer) over time as a proxy of cell division. Axenicity of diatom cultures was monitored by inoculating them into marine broth (MB; 1:10 dilution) before every experiment to confirm the absence of bacterial contamination. *A. macleodii* EP glycerol stocks and other bacteria tested in this study (Table S3) were streaked on marine agar plates (MB) containing 1.5% (wt/vol) agar and grown at 25°C for 48 h. Single colonies were grown overnight in MB with shaking at 25°C until an OD_600_ of ~0.5 was reached (NanoDrop One Microvolume UV-Vis Spectrophotometer). The resulting bacterial cultures were diluted 1:10 and allowed to double twice (~3:30 h) before removing the media and washing twice with L1 + Si. The washed *A. macleodii* cells were added to a diatom culture at a final concentration of ~40,000 cells/mL, corresponding to an inoculum volume of 1–10 µL in a 25 mL culture. All experiments were performed in L1 + Si medium or L1 + Si medium supplemented with 2% MB (vol/vol), either at the beginning of the co-culture or at a specified time point. Analyses relied on two biological replicates, a factor that limits the scope of inference.

### Exudate experiments

Samples were grown as previously described, and exudates were collected by filtering twice through a 0.22 µm glass fiber filter at the specified time points. Bacterial cells were cultured as described above and then inoculated into the exudates at a concentration of 40,000 cells/mL, using the same volume ratio as in the co-cultures. Diatoms from exponentially growing cultures were diluted into the various exudates at 2% (vol/vol), initiating the experiments with an initial concentration of approximately 200,000 cells/mL.

### Flow cytometry

Samples were incubated with SYBR Green (1:5,000) for 30 min at room temperature in the dark, followed by analysis using a Guava easyCyte 11HT Benchtop Flow Cytometer. *T. pseudonana* cells were detected based on chlorophyll autofluorescence (emission at 642 nm) and forward scatter, while *A. macleodii* EP cells were detected by SYBR fluorescence (emission at 532 nm) and forward scatter. Gating parameters were established using axenic cultures for each species.

### Nutrient [PO_4_, Si(OH)_4_, NO_3_, NO_2_, and NH_4_] measurements

Samples were filtered through a 0.22 µm glass fiber filter, immediately frozen, and were analyzed at the UW Marine Chemistry Laboratory with a Seal Analytical AA3, following the protocols of the WOCE Hydrographic Program (67).

### Dissolved organic carbon (DOC) measurements

Samples were filtered using 25 mm carbon-cleaned GF/F filters (combusted at 450°C for 4 h), immediately frozen, and were analyzed at the UW Marine Chemistry Laboratory with a Shimadzu TOC-Vcsh DOC analyzer, following the protocols of the WOCE Hydrographic Program (67).

### RNA extraction and library preparation

Total RNA was extracted from 0.2 µm polycarbonate membrane filters using the Zymo Direct-zol RNA MiniPrep Plus kit for all samples. For *T. pseudonana* transcriptome ribodepletion calibration, three samples obtained from exponentially growing cells were prepared: total RNA, poly(A) selected with oligo dT-beads (Dynabeads mRNA DIRECT Kit, Life Technologies), and ribodepleted using a tailored Thalassiosira pseudonana riboPOOL following the manufacturer’s instructions. For Duo-RNA-seq, samples were prepared in triplicate for each time point and ribodepleted using the *T. pseudonana* riboPOOL, designed based on an updated version of the rRNA sequences (68) combined with the Pan-Bacteria riboPOOL, with beads mixed in a 1:1 ratio. At this point, libraries were submitted for preparation and sequencing to the Northwest Genomics Center (University of Washington) using a NextSeq (Illumina) platform.

### Ribo-depletion efficiency calculation

Sequence reads were trimmed using Trimmomatic 0.39 (69), run in paired-end mode with the adaptor and other Illumina-specific sequences (ILLUMINACLIP) set to TruSeq3-PE.fa:2:30:10:1, leading and trailing quality thresholds of 25, a sliding-window trimming approach (SLIDINGWINDOW) of 4:15, an average quality level (AVGQUAL) of 20, and a minimum length (MINLEN) of 60. For RNA identification, paired-end reads were aligned with STAR v2.7.10b (70) to the *T. pseudonana* genome (Thaps3, FilteredModels2) (71), plus an additional “chromosome” containing an updated version of the ribosomal sequences determined using Nanopore sequencing (68). Default parameters were used, with the maximum intron length limited to 500 nucleotides (“–alignIntronMax 500“). Reads that aligned to both the *T. pseudonana* genome and the rRNA chromosome were assigned to the latter. Reads per chromosome were calculated using the samtools idxstats command (72) and assigned to gene models using the featureCounts tool (73). The rRNA read fraction was calculated as the total number of reads assigned to the rRNA chromosome divided by the total aligned reads. The gene model read fraction was calculated as the total number of reads assigned to a gene model divided by the total number of aligned reads. The tailored kit effectively minimized host rRNA representation in the sequence data (Fig. S4B and C).

### *A. macleodii* genome determination

To determine the most similar genome option, we first selected all *A. macleodii* genomes available in the NCBI genome database that met the following criteria: (i) complete assembly level, (ii) annotated, (iii) not from metagenome-assembled genomes (MAGs), and (iv) not labeled as atypical. This resulted in a total of 14 genomes (Table S4). Paired-end reads were analyzed as previously described but mapped to the 14 genomes in parallel. To determine which genome best matched the *A. macleodii* EP strain, we used three metrics: the percentage of uniquely aligned reads, the fraction of reads assigned to gene models, and the number of gene models with more than five reads. We confirmed the ability to distinguish *A. macleodii* and *T. pseudonana* reads by analyzing the axenic cultures, which showed no bacterial reads, and by detecting an increase in bacterial reads over time, particularly in the stationary-phase co-culture (Fig. S4E).

### Duo-RNA-seq analysis

Paired-end reads were analyzed as previously described, but mapped to a combined *T. pseudonana* (Thaps3, FilteredModels2) and *A. macleodii* Te101 genome (25, 71). Gene counts for each genome were processed separately, with trimmed mean of M-values (TMM) normalization and RPKM calculation performed using the EdgeR package (74). The batch effect from different rounds of ribodepletion was removed using the ComBat function (75). For PCA analysis, counts were scaled and then analyzed using the R function prcomp. Differential expression analysis was conducted using the limma package (76) for the specific contrasts. To identify significant Gene Ontology (GO) terms associated with differentially expressed genes, we employed the topGO R package (77) using GO annotations provided by Hou et al. (25). Additionally, KEGG Orthology (KO) IDs for each gene model were identified using the BlastKOALA tool (78), and selected pathways were retrieved manually. Additionally, enrichment of KOG categories among *T. pseudonana* differentially expressed genes was tested using a proportion test (chi-squared test for equality of proportions) against their distribution in the full transcriptome.

### *A. macleodii* MIT1002 co-culture with *Prochlorococcus* and *A. macleodii* ATCC 27126 monoculture RNA-seq analysis

An identical pipeline was used as in Duo-RNA-seq. To assess conservation, the total number of gene reads across all samples was summed, and a threshold of 50 reads was reported (similar trends were obtained with thresholds of 10 and 100 reads).

### Fluorescent microscopy

Two hundred microliters of *T. pseudonana* cultures or co-cultures with *A. macleodii* EP was stained with SYBR Green (1:5,000) and incubated for 30 min at room temperature in the dark. After incubation, samples were centrifuged at 10,000 rpm for 1 min, and the supernatant was discarded, leaving a final volume of 10 µL. The concentrated samples were then mounted onto transparent slides, and images were captured at 20× magnification using a Leica DMi8 microscope. Fluorescent images were taken for DNA (green channel), chlorophyll (red channel), and brightfield. Image processing was performed using ImageJ (79).

### Casein plate assay

Casein agar plates were prepared in small batches. For every 100 mL of artificial seawater, 0.3 g of casein protein was well dissolved. After full dissolution, 0.2 g of yeast extract and 1.5 g of Bacto-agar were added. After autoclaving, plates were stored in the fridge until ready for use. Two biological replicates and two technical replicates were prepared for the following conditions: diatom exudate (*T. pseudonana* grown 24 h in L1 + Si medium), co-culture exudate (*T. pseudonana* and *A. macleodii* grown 24 h in L1 + Si medium), bacterial exudate (*A. macleodii* grown 24 h in L1 + Si medium supplemented with 2% MB [vol/vol]), and co-culture + MB exudate (*T. pseudonana* and *A. macleodii* grown 24 h in L1 + Si medium supplemented with 2% MB [vol/vol]). Growth conditions and filtration were performed as previously described. Immediately after filtration, 7 μL droplets of each condition were placed onto marked sections of the casein agar plates. Plates were stored in the same conditions as the co-culture experiments. The diameter of each halo was measured 6 days after plating.

### Statistical analysis

To evaluate the effects of experimental factors on the cell numbers of either *T. pseudonana* or *A. macleodii*, while accounting for variability across replicates, we applied a linear modeling approach in R. Replicate identity was treated as a fixed effect, rather than as a random effect, to account for systematic differences between replicates due to the limited number of biological replicates. Fixed effects included the relevant experimental conditions and their interactions. An analysis of variance (ANOVA) was performed on the linear model to assess the significance of the fixed effects. To assess robustness to replicate structure, we performed permutation tests by randomly shuffling replicate labels. The observed F-statistic was not extreme relative to the null distribution, indicating that the tested effect was not supported and did not depend on replicate assignment. *Post hoc* pairwise comparisons were conducted using estimated marginal means (EMMs) with Bonferroni-adjusted *P*-values to control for multiple testing, as implemented via the emmeans package (80) (Table S5).

## Data Availability

The data sets generated in this study for RNA expression are available on GEO under the accession number GSE291849. *A. macleodii* MIT1002 co-culture with *Prochlorococcus* RNA expression data were obtained from GEO (accession number GSE73511). *A. macleodii* ATCC 27126 nutrient scarcity RNA expression data were obtained from NCBI BioProject number PRJNA591216.

## SUPPLEMENTAL MATERIAL

Data S1 mbio.00275-26-s0001.pdf Merged forward and reverse Sanger 16S sequences for *A. macleodii* EP.

Supplemental Figures mbio.00275-26-s0002.pdf Figures S1 to S10.

Captions mbio.00275-26-s0003.pdf Captions for supplemental tables and Data S1.

Table S1 mbio.00275-26-s0004.xlsx Blast results of comparing *A. macleodii* 16S to NCBI references.

Table S2 mbio.00275-26-s0005.xlsx Differentially expressed genes in *T. pseudonana*.

Table S3 mbio.00275-26-s0006.xlsx Species used in this study.

Table S4 mbio.00275-26-s0007.xlsx *A. macleodii* genomes.

Table S5 mbio.00275-26-s0008.xlsx Linear model results assessing the effects of experimental factors on *T. pseudonana* and *A. macleodii* cell numbers.

ASM does not own the copyrights to Supplemental Material that may be linked to, or accessed through, an article. The authors have granted ASM a non-exclusive, world-wide license to publish the Supplemental Material files. Please contact the corresponding author directly for reuse.
