# Supplementary material for: Thalassiosira pseudonana growth phase determines gene expression and algicidal behavior of a new Alteromonas macleodii strain
Source: mBio. 2026 Mar 23;17(4):e00275-26. doi: 10.1128/mbio.00275-26 (PMC13059764; doi:10.1128/mbio.00275-26)
Supplement: Supplemental Figures — Figures S1 to S10. [file mbio.00275-26-s0002.pdf]

Supplementary Figures:

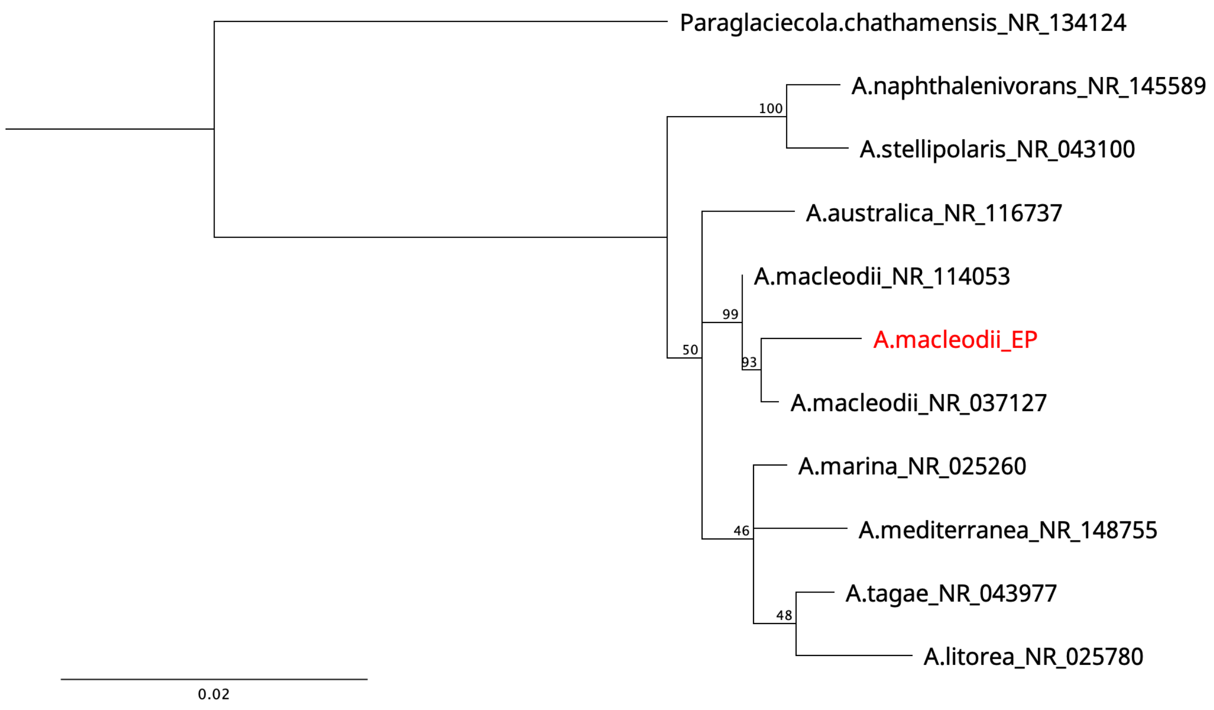

Supp 1. New Equatorial Pacific bacterial strain clusters within *A. macleodii* species. A maximum likelihood 16S rRNA phylogenetic tree of *A. macleodii* EP (in red) and the closest relatives based on an NCBI BLAST search. The tree is rooted in the outgroup *Paraglaciecola chathamensis*, and branches are labelled by bootstrap consensus support percent.

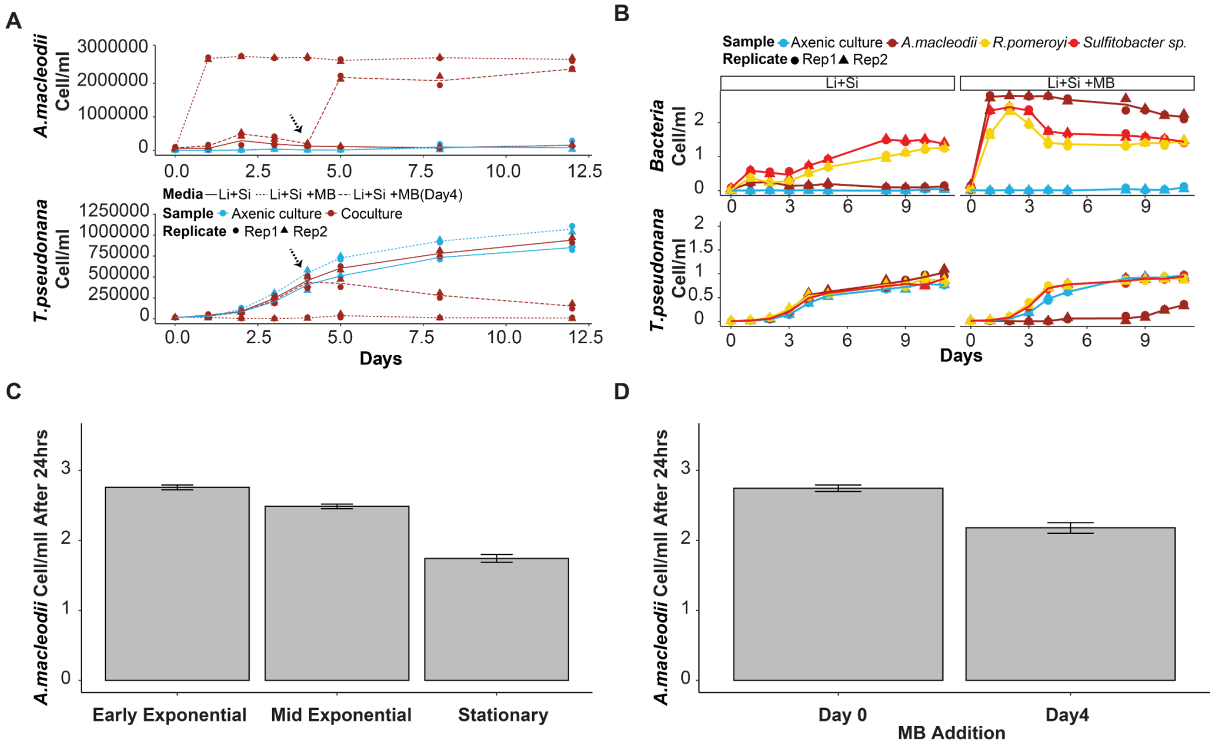

Supp 2. *T. pseudonana* growth phase and substrate supply determine interaction dynamics with *A. macleodii* EP. (A) Similar to **Fig. 1A**, showing cell counts in co-culture (magenta symbols) or mono-culture (blue symbols). Dashed lines indicate the timing of early and late MB addition. The arrow indicates MB addition, and the line is the average between two replicates. **(B)** As in **Fig. 1A,** each facet represents cultures grown in L1+Si with or without MB supplementation. Colors indicate specific cocultures: *A. macleodii* (magenta), *R. pomeroyi* (yellow), *Sulfitobacter* sp. (red), and axenic culture (blue symbols), and the line is the average between two replicates. **(C)** Bar plot showing bacterial cell counts (cells/mL) 24 hours after coculture initiation across different diatom growth phases. **(D)** As in (C), comparing early vs. late MB addition to the coculture.

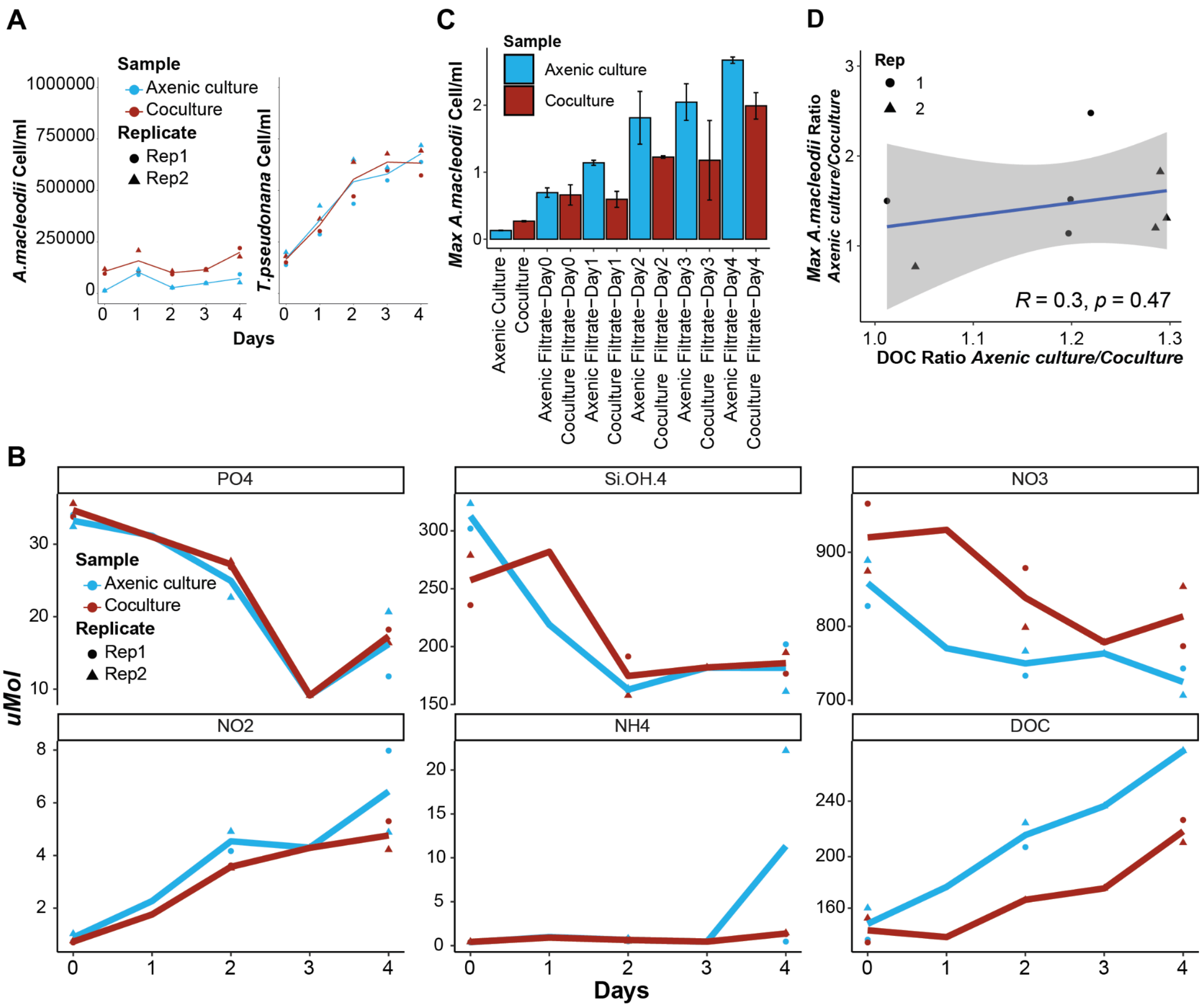

Supp 3. *T. pseudonana* imposes constraints on bacterial growth beyond nutrient limitation. (A) Similar to **Fig. 1A**, at the mid-exponential stage, exudates were collected from the axenic culture and the co-culture every 24h. **(B)** Scatter plots showing the selected nutrient concentrations of the corresponding day's exudates. Color code distinguishes axenic cultures from cocultures. **(C)** Bar plot showing maximal bacterial cell counts (cells/mL) in the original samples and in each exudate. The error bar shows the standard deviation, and the color code distinguishes axenic culture from coculture samples. **(D)** Scatter plot between the ratio of maximal bacterial cell counts between the Axenic culture and Coculture, plotted against the DOC ratio. The Pearson correlation coefficient and associated p-value are included.

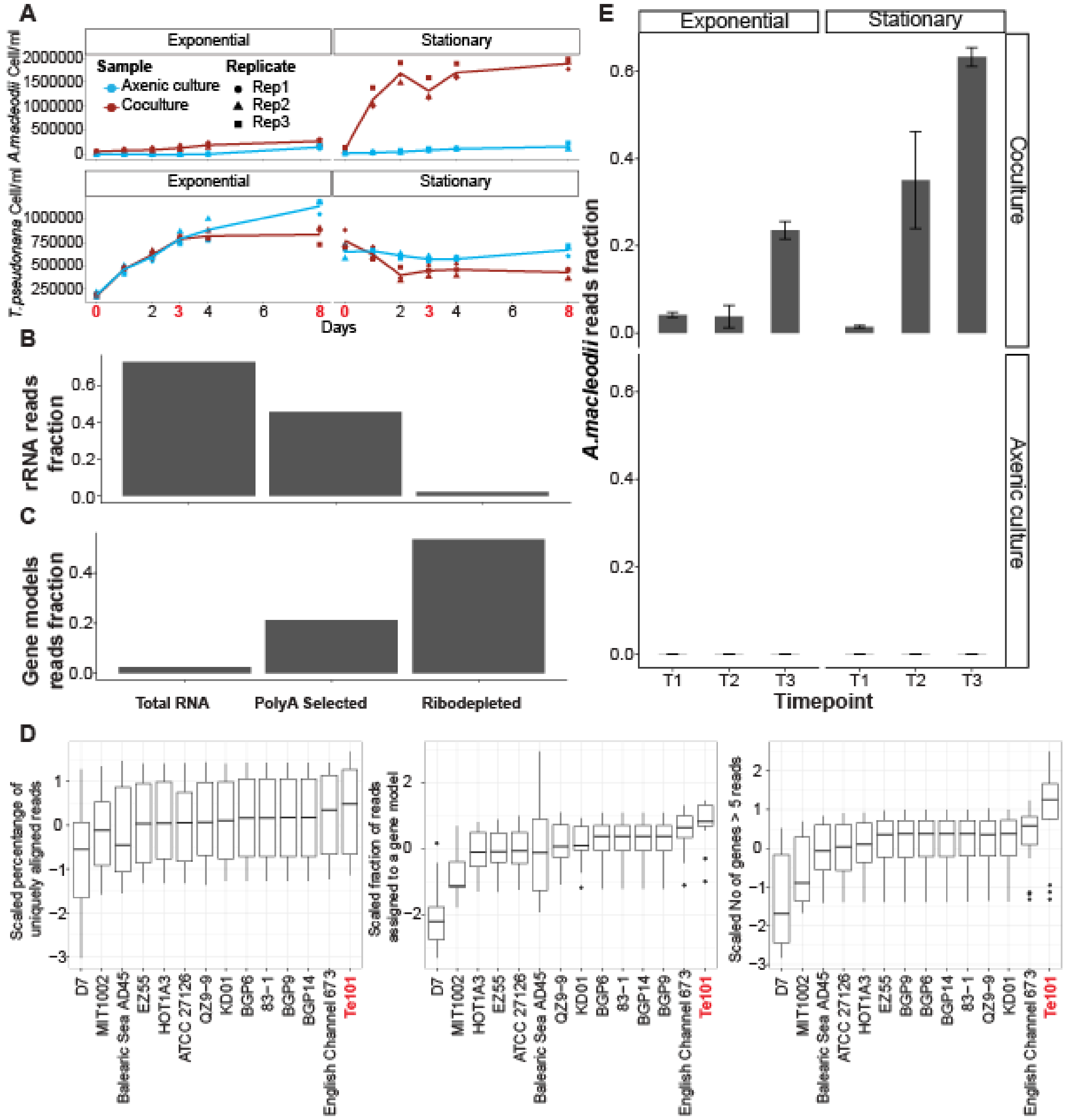

Supp 4. Tailored Duo-RNAseq protocol efficiently removes *T. pseudonana* rRNA and accurately assigns reads to the corresponding genome. (A) As in **Fig. 1A,** days marked in red indicate the sampling timepoints for Duo-RNAseq, which are from 3 biological replicates. **(B)** Barplot showing the rRNA reads fraction in three different *T. pseudonana* RNA library preparation protocols (Total RNA, Poly(A) selection, and Ribodepletion). **(C)** Similar to A, but for the gene models' read fraction. **(D)** Boxplot showing the percentage of uniquely aligned reads (left), the fraction of reads assigned to a gene model (middle), and the number of gene models with more than five reads for all coculture samples per *A. macleodii* genome (Table 2). Each metric was scaled across coculture time points. The selected genome is highlighted in red. **(E)** Barplot showing the fraction of A. macleodii EP-assigned reads at each time point. Error bars represent the standard deviation of three replicates. Facets distinguish between Axenic culture and Coculture, as well as Stationary and Exponential samples.

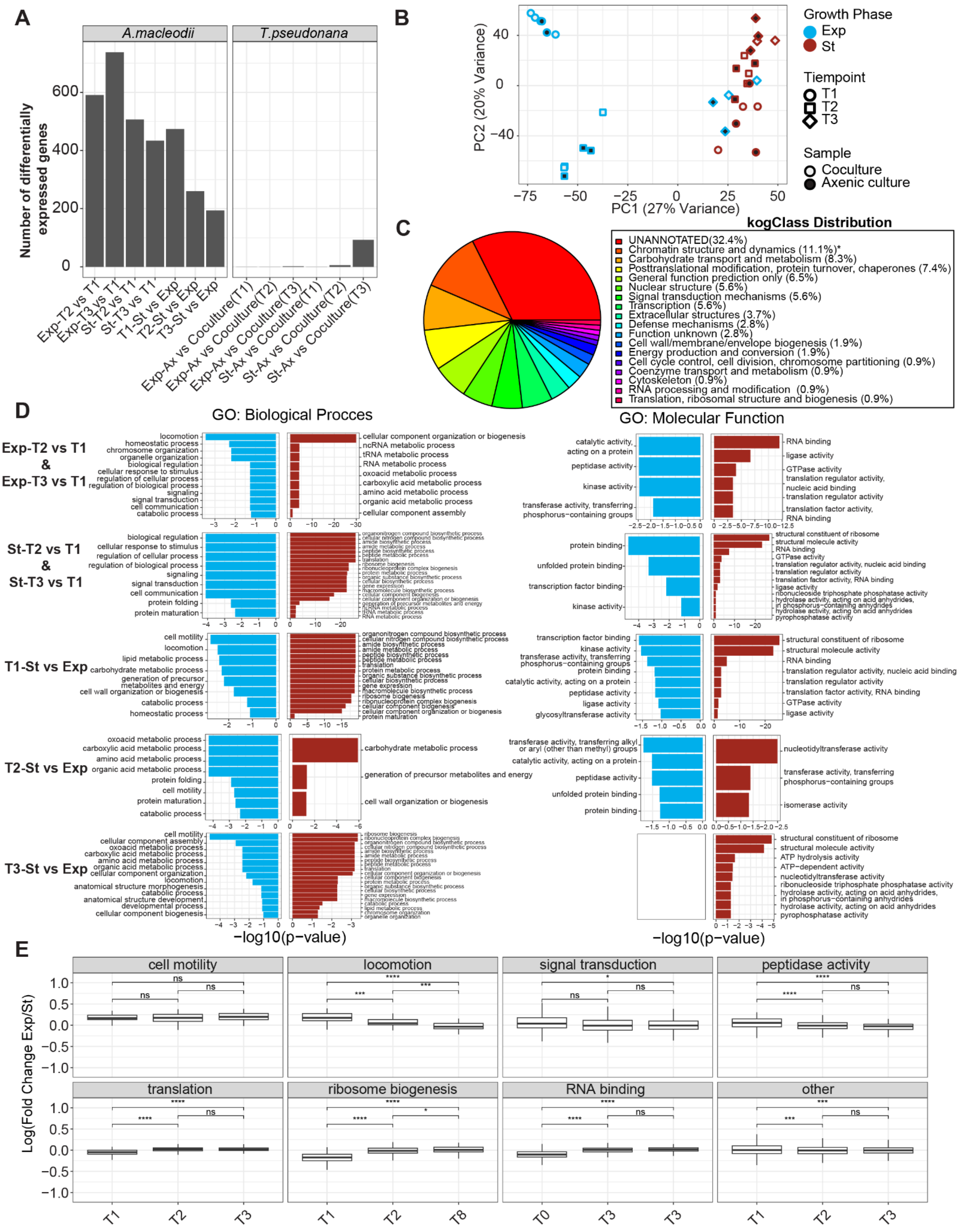

Supp 5. Gene expression regulatory landscape in the *A. macleodii* EP–*T. pseudonana* interaction. (A) Bar plot showing the number of differentially expressed genes in both genomes for specific comparisons**. (B)** Principal component analysis (PCA) using scaled RPKM reads from *T. pseudonana*. The internal color code corresponds to the axenic culture (black) and coculture (white), while the external color code represents the initial diatom growth phase (exponential - blue, stationary - magenta). Shapes represent the time points: Circles for T1, Squares for T2, and Triangles for T3. **(C)** Pie chart showing the distribution of KOG class categories among differentially expressed genes for the *T. pseudonana* genome. * indicates statistically significant enrichment compared to the full transcriptome (Chi-squared test for proportions). **(D)** Gene ontology terms associated with significantly upregulated (magenta) or downregulated (blue) genes in the indicated comparisons. **(E)** Log fold

change between Exponential and Stationary for the selected GO at each time point. Significance of the Wilcoxon signed-rank test p-values is indicated for each comparison.

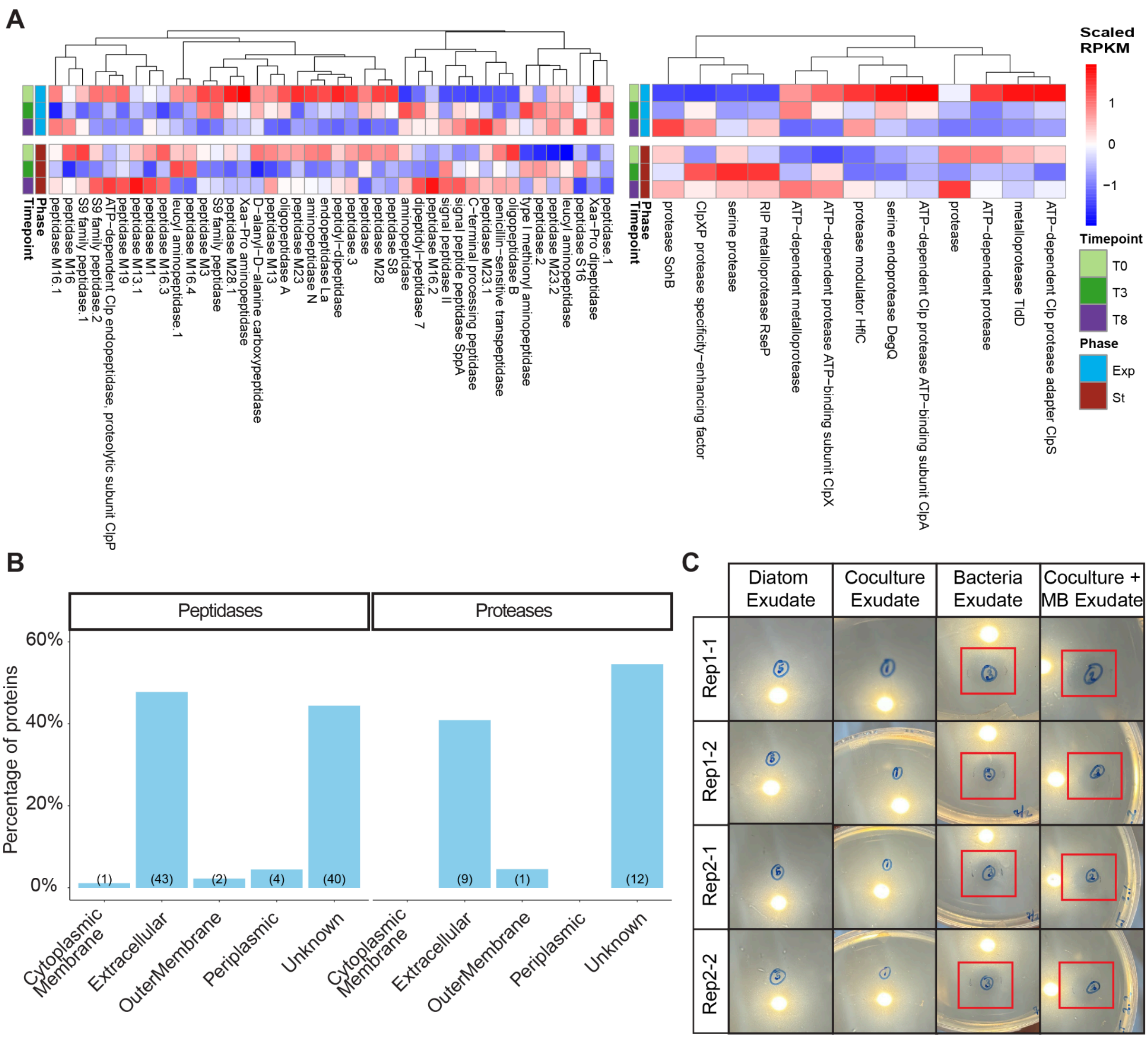

Supp. 6. *A. macleodii EP* secretes peptidases and/or proteases during the coculture. (A) Heatmaps of gene expression for *A. macleodii* peptidases (left) and proteases (right). The color scale represents scaled TMM-normalized RPKM values. Columns (genes) were clustered using Euclidean distance with the pheatmap function in R. Row annotations indicate sample type: exponential phase (blue) and stationary phase (magenta). Time points are marked in light green (T1), green (T2), and purple (T3). For genes with identical annotations, a dot and a number were appended to the gene name. **(B)** Bar plot showing PSORTb localization prediction of peptidases (left) and proteases (right) encoded in the *A. macleodii* Te101 genome. The y-axis represents the percentage of proteins in each localization category, and the numbers in parentheses indicate the absolute number of proteins per localization. **(C)** Table showing photographs of the casein agar plates after 6 days had elapsed. The left legend indicates the replicant group, while the top legend indicates the condition. Halos (clear circles) indicate the presence of proteases; the halos are highlighted with red boxes.

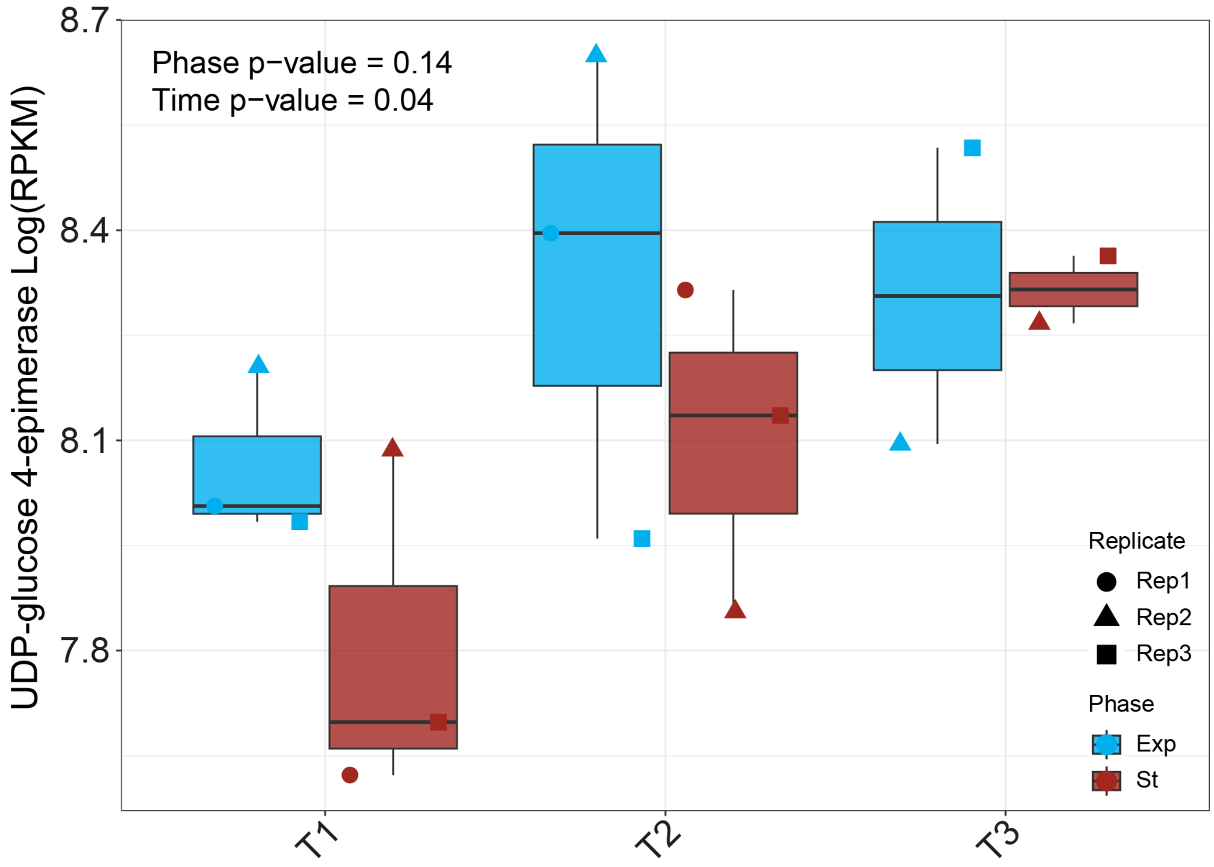

Supp. 7. *UDP-glucose 4-epimerase* transcript levels increase with time in coculture. Boxplots showing the RPKM values of the *A. macleodii* gene *UDP-glucose 4-epimerase* in exponential (blue) and stationary (magenta) phases of diatom cocultures. Each biological replicate is represented by a dot with a distinct shape.

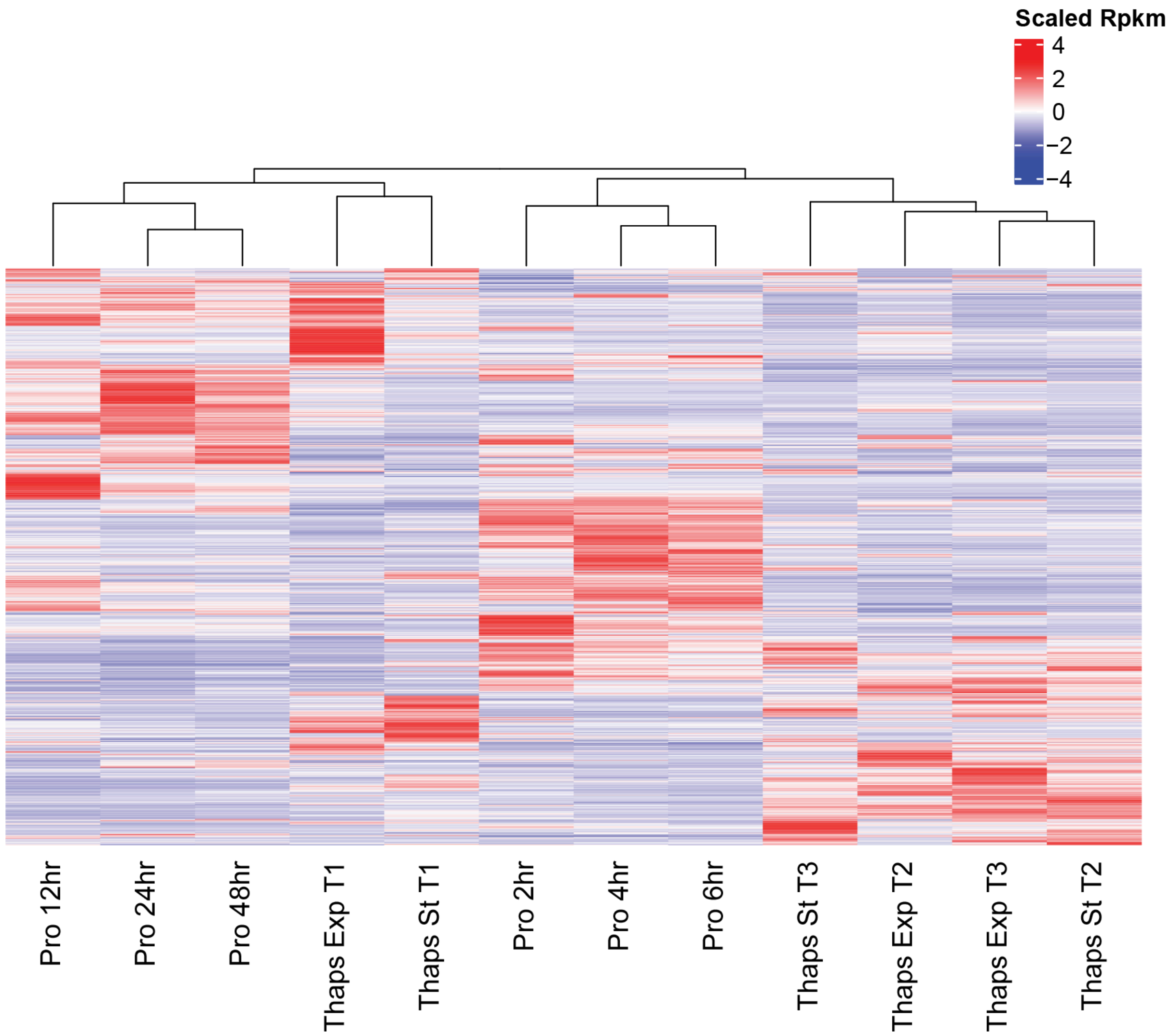

Supp. 8. *A. macleodii* Expression profiles cluster according to time in coculture and host. Heatmap of gene expression across both co-cultures. The color scale represents scaled TMM-normalized RPKM values. Rows and columns were clustered using Euclidean distance with the pheatmap function in R.

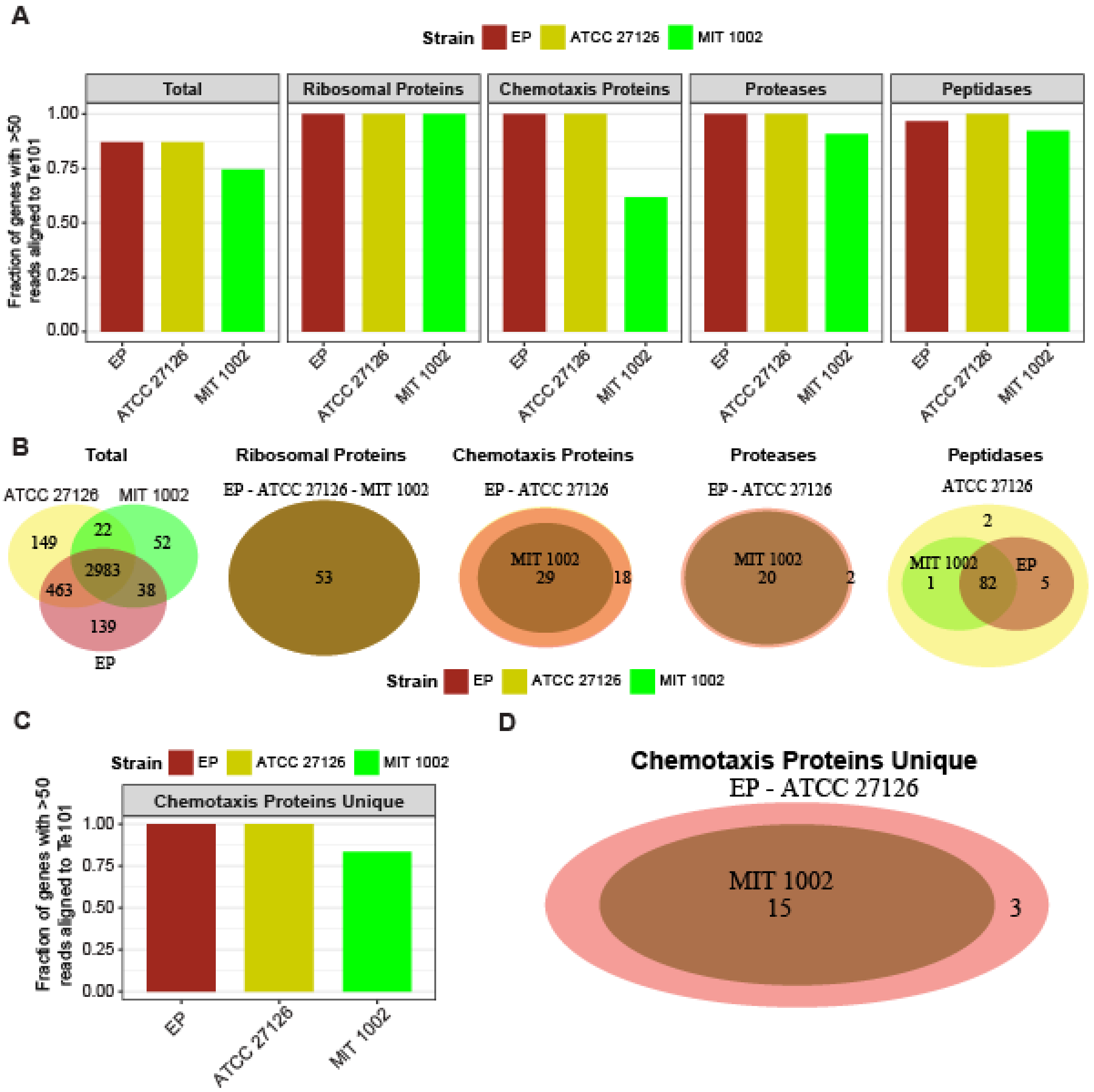

Supp. 9. Gene presence largely overlaps between strains compared in this study. (A) Bar plot showing the number of genes with more than 50 reads aligned to the Te101 genome across all samples from this study, Mank et al., and Biller et al. Colors indicate the different strains used in each study. **(B)** Venn diagram showing the overlap of genes with more than 50 reads between the studies. **(C)** Analysis similar to (A, left) and (B, right), after aggregating reads per gene annotation for chemotaxis proteins.

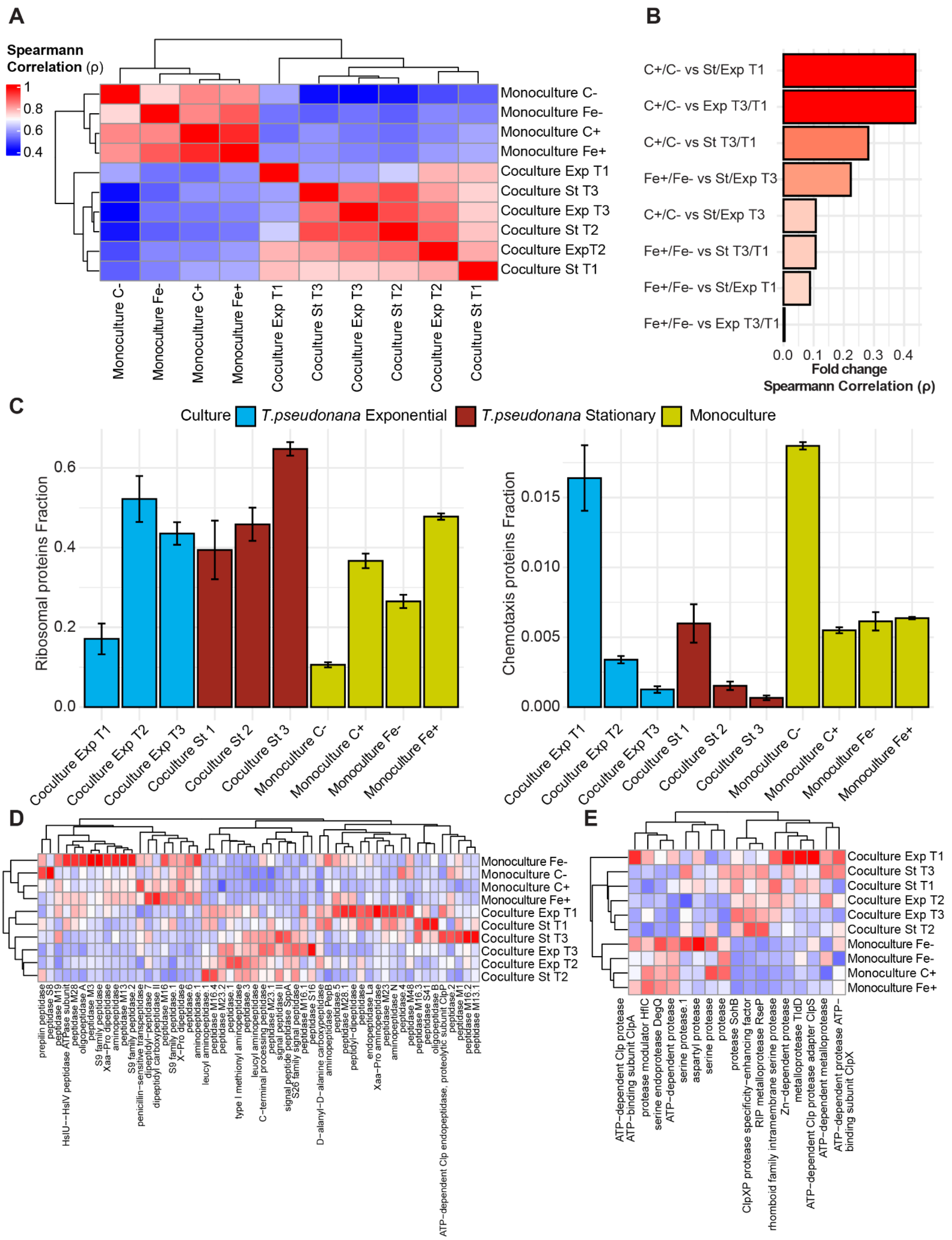

Supp. 10. Coculture expression progression partially overlaps with nutrient-driven changes (A) Heatmap showing Spearman correlations between the average TMM-normalized RPKM values of three replicates per treatment. Monoculture samples were obtained from Mank *et al.* **(B)** Bar plot showing the correlation between fold-change differences observed under nutrient limitation treatments (Fe and C; Mank *et al.*) and the fold changes across time (T3/T1) and growth stage (St/Exp). The color code indicates the intensity of the correlation. **(C)** Bar plots showing the fraction of ribosomal proteins (left) and chemotaxis proteins (right) in co-cultures and monocultures. Error bars represent the standard deviation between two or three replicates. **(D-E)** Heatmaps of gene expression for *A. macleodii* peptidases (left) and proteases (right)**.** The color scale represents scaled

TMM-normalized RPKM values. Columns (genes) were clustered using Euclidean distance with the pheatmap function in R.
