# Supplementary material for: Thalassiosira pseudonana growth phase determines gene expression and algicidal behavior of a new Alteromonas macleodii strain
Source: mBio. 2026 Mar 23;17(4):e00275-26. doi: 10.1128/mbio.00275-26 (PMC13059764; doi:10.1128/mbio.00275-26)
Supplement: Captions — for supplemental tables and Data S1. [file mbio.00275-26-s0003.pdf]

Supplemental Table 1: Blast results of comparing *A. macleodii* 16S to NCBI references. Top 30 references based on e-value and similarity score.

Supplemental Table 2: List of differentially expressed genes in *T. pseudonana.*

Supplemental Table 3: Species used in this study.

Supplemental Table 4: *A. macleodii* genomes list.

Supplemental Table 5: Linear model results assessing the effects of experimental factors on T. pseudonana and A. macleodii cell numbers, with replicate treated as a fixed effect. The table includes ANOVA F-statistics and p-values, F-statistics obtained from permutation tests with randomly shuffled replicate labels, Bonferroni-adjusted post hoc pairwise comparisons based on estimated marginal means, and bootstrap-derived mean estimates with 95% confidence intervals (500 iterations) for both ANOVA terms and post hoc contrasts.

Supplemental Data 1: Merged forward and reverse Sanger 16S sequences for *A. macleodii* EP.
